# Overexpression of the cohesin-core subunit *SMC1A* contributes to colorectal cancer development

**DOI:** 10.1186/s13046-019-1116-0

**Published:** 2019-03-01

**Authors:** Patrizia Sarogni, Orazio Palumbo, Adele Servadio, Simonetta Astigiano, Barbara D’Alessio, Veronica Gatti, Dubravka Cukrov, Silvia Baldari, Maria Michela Pallotta, Paolo Aretini, Felice Dell’Orletta, Silvia Soddu, Massimo Carella, Gabriele Toietta, Ottavia Barbieri, Gabriella Fontanini, Antonio Musio

**Affiliations:** 1 Institute for Genetic and Biomedical Research (IRGB), National Research Council (CNR), Via Moruzzi, 1, 56124 Pisa, Italy; 2 Division of Medical Genetics, IRCCS “Casa Sollievo della Sofferenza”, San Giovanni Rotondo, Italy; 3 Division of Pathology, Department of Surgery, University of Pisa, Pisa, Italy; 4 IRCCS Ospedale Policlinico San Martino, Department of Translational Oncology, Genoa, Italy; 5 IRCCS Regina Elena National Cancer Institute, Department of Research, Advanced Diagnostic and Technological Innovation, Rome, Italy; 6 Fondazione Pisana per la Scienza ONLUS, San Giuliano Terme, Italy; 7 Institute for Computational Linguistics (ILC) “A. Zampolli”, National Research Council (CNR), Pisa, Italy; 8 Department of Experimental Medicine, University of Genoa, Genoa, Italy; 9 Present address: Institute of Cell Biology and Neurobiology, National Research Council (CNR), Monterotondo, Italy

**Keywords:** Cohesin, SMC1A, Chromosome instability, Gene expression dysregulation, Human colorectal cancer development

## Abstract

**Background:**

Cancer cells are characterized by chromosomal instability (CIN) and it is thought that errors in pathways involved in faithful chromosome segregation play a pivotal role in the genesis of CIN. Cohesin forms a large protein ring that binds DNA strands by encircling them. In addition to this central role in chromosome segregation, cohesin is also needed for DNA repair, gene transcription regulation and chromatin architecture. Though mutations in both cohesin and cohesin-regulator genes have been identified in many human cancers, the contribution of cohesin to cancer development is still under debate.

**Methods:**

Normal mucosa, early adenoma, and carcinoma samples deriving from 16 subjects affected by colorectal cancer (CRC) were analyzed by OncoScan for scoring both chromosome gains and losses (CNVs) and loss of heterozygosity (LOH). Then the expression of SMC1A was analyzed by immunochemistry in 66 subjects affected by CRC. The effects of *SMC1A* overexpression and mutated *SMC1A* were analyzed in vivo using immunocompromised mouse models. Finally, we measured global gene expression profiles in induced-tumors by RNA-seq.

**Results:**

Here we showed that *SMC1A* cohesin core gene was present as extra-copies, mutated, and overexpressed in human colorectal carcinomas. We then demonstrated that cohesin overexpression led to the development of aggressive cancers in immunocompromised mice through gene expression dysregulation.

**Conclusion:**

Collectively, these results support a role of defective cohesin in the development of human colorectal cancer.

**Electronic supplementary material:**

The online version of this article (10.1186/s13046-019-1116-0) contains supplementary material, which is available to authorized users.

## Background

Chromosomal instability (CIN) is characterized by chromosome translocations, allelic imbalances and aneuploidy. It is thought that CIN is an early step during tumorigenesis that allows cells to acquire additional genetic changes required for full malignant transformation. There have been extensive studies on the causes and consequences of CIN because it is a characteristic of many cancer cells.

Cohesin is an evolutionarily conserved four-subunit (SMC1A, SMC3, RAD21, and either STAG1 or STAG2) complex that encircles DNA within its ring-shaped structure. It was first identified for its role in ensuring sister chromatid cohesion, which is essential for correct chromosome segregation [1]. The cohesin core complex interacts with several regulatory-cohesin factors contributing to its function including NIPBL, required for the loading of cohesin onto chromatin, ESCO2, essential for the establishment of physical bridges between sisters during S phase, PDS5A and PDS5B, which interact with cohesin for its establishment and maintenance and HDAC8, required for the removal of cohesin [2–8].

Increasing evidence suggests that cohesin also participates in many additional biological processes. Indeed, it promotes DNA repair by homologous recombination and non-homologous end joining [9–11], favors the recruitment of proteins involved in the activation of the intra-S and G2/M checkpoints [12, 13], controls fork replication speed [14, 15] and regulates gene transcription by mediating functional connections between promoters and their distal enhancers [16, 17].

Germline mutations in cohesin and its regulators are responsible for Cornelia de Lange syndrome (CdLS) [18–23], a rare developmental disease characterized by both chromosome aneuploidies and aberrations, precocious sister chromatid separation and sensitivity to genotoxic drugs [24–27]. Despite these considerations, CdLS patients do not show predisposition to cancer [26]. Instead, somatic mutations in the *NIPBL*, *RAD21*, *SMC1A*, *SMC3* and *STAG2* genes have been described in many human cancers including acute myeloid leukemia, bladder, and colorectal cancer [28–33]. Other cohesin and regulatory-cohesin genes are not frequently mutated in cancer. The mechanism through which mutated cohesin promotes tumorigenesis is still controversial. *STAG2* is significantly mutated in four or more human cancer types [33] and experimental data indicates that *STAG2* gain-of-function changes cause loss of sister chromatid cohesion and CIN [31, 32, 34, 35]. However, data obtained in naturally occurring tumors suggests no correlation between cohesin mutations and CIN [36]. In addition, *STAG2*-deficient tumors are often euploid [36, 37]. Therefore, the significance of these mutations in promoting tumorigenesis remains debatable and elusive. These conflicting data are likely in part attributable to the fact that cohesin plays a critical dual role in both gene transcription regulation and maintenance of genome stability. Furthermore, mutational screening has been performed only at the carcinoma stage and no study has been conducted at different stages during tumorigenesis.

Human colorectal cancer (CRC) is an important contributor to cancer morbidity and mortality worldwide. It is classified as microsatellite instability (15% of cases, associated with a better prognosis) and chromosomally unstable (85% of affected patients, with a worse prognosis) [38, 39]. In an effort to provide new insight into the role of cohesin in tumorigenesis, we analyzed the copy number variations (CNVs) by OncoScan in normal mucosa, early adenomas, and carcinomas derived from 16 subjects affected by CRC. We found extra-copies of *SMC1A* gene in carcinomas and subsequent sequencing revealed the presence of mutations. Furthermore, the analysis of SMC1A expression in a larger cohort of patients (*n* = 66) showed that it is significantly more overexpressed in carcinomas than in both early adenomas and normal mucosa. Finally, we demonstrated that cohesin overexpression produces aggressive tumors in nude mice through gene expression dysregulation. Collectively, these findings identify *SMC1A* as a contributing gene in CRC development and may serve as a promising therapeutic strategy.

## Methods

### OncoScan analysis

Normal mucosa, early adenoma, and carcinoma from 16 subjects affected by CRC (Additional file 1: Table S1) were processed for identification of copy number imbalances through Molecular Inversion Probe (MIP) based OncoScan Array following protocols provided by the manufacturer (Affymetrix). DNA samples were normalized to 12 ng/μL, mixed with MIPs and incubated overnight to anneal (16–18 h). Next, each reaction was divided equally into A and B reactions and “Gap Fill” master mix was added with either AT dNTPs (A reaction) or GC dNTPs (B reaction) and incubated. Following the “Gap Fill” reaction, exonuclease was added to remove unligated probes and genomic DNA. Next, MIPs were linearized with a restriction enzyme and PCR amplified (PCR 1). Reactions were taken through a second round of amplification (PCR 2), and subsequently digested with HaeIII restriction enzyme. The digested products were hybridized to the OncoScan Array at 58 °C for 16–18 h. Arrays were stained and washed using the GeneChip Fluidics Station 450 and loaded on the GeneChip Scanner 3000 7G (Affymetrix) where fluorescence intensity was scanned to generate array images (DAT files).

Next, array fluorescence intensity data (CEL) files were generated with an Affymetrix GeneChip Command Console (AGCC, Affymetrix) and used to produce OSCHP files and QC metrics through OncoScan Console Software (Biodiscovery). The standard Affymetrix reference control file for OncoScan data was used for processing the arrays. Samples passing QC criteria (MAPD ≤0.3, ndSNPQC ≥26) were further analyzed using tumor Scan (TuScan) and BioDiscovery’s SNPFASST2 algorithm using the Nexus Express for OncoScan software version 3.0 and 7.5 (Biodiscovery). The TuScan algorithm creates segmentation to differentiate between adjacent clusters of probes and determines the copy number changes. SNP-FASST2 uses a Hidden Markov Model (HMM)-based approach to identify larger copy number segments based on a log-ratio threshold derived from all probes in a given region. Ratios are the log2 ratios of the normalized intensity of the sample over the normalized intensity of a reference with further correction for a sample-specific variation. The Median Log2 Ratio was computed for each segment detected in the analysis. The significance threshold for segmentation was set at 1.0e − 5 also requiring a minimum of three probes per segment and a maximum probe spacing of 1000 kbp between adjacent probes before breaking a segment. Segments were classified as having gains when the Log2Ratio (L2R) exceeded 0.2, losses when L2R was < − 0.2, and with high copy gains and homozygous losses being called when L2R was > 0.6 and < − 1.0, respectively. Differences in copy number changes between samples from a tumor for individual genes were counted when one sample had a change as defined above and another either did not have that change or had a different change. B-allele frequency (BAF) information was also generated for each tumor. BAF is a normalized measure of the allelic intensity ratio of two alleles (A and B), such that a BAF of 1 or 0 indicates the complete absence of one of the two alleles (e.g. AA or BB), and a BAF of 0.5 indicates the equal presence of both alleles (e.g. AB). Median BAF is reported for each segment and is the median BAF of the markers identified as heterozygous. If the number of heterozygous markers in the segment is below 10 or the percent of homozygous markers is above 85% no value is reported. The B-allele frequency values are used to determine whether a segment is in a loss of heterozygosity (LOH) or an allelic imbalance state. By default, probe sets were automatically centered to the median for all samples by the Nexus software. For individual samples where the median probe set value was not diploid, specified regions of balanced heterozygosity were manually identified by visual inspection of L2R and BAF plots and defined as diploid regions, permitting the Nexus software to reset the entire probe set to the newly defined areas [40]. Finally, we carefully analyzed the CNVs involving the X chromosome. In detail, we used a different threshold for the CNVs of the X chromosome in males and females (Copy number = 0, for a deletion identified in a male; Copy number = 2 or greater than 2, for duplications identified in a male subject. Copy number = 1 or 0 for a deletion in a female; Copy number = 3 or greater than 3 for a duplication in a female subject).

### Clinical samples and histopathology

Sixty-six subjects affected by CRC (Additional file 1: Table S2) were retrospectively selected from the files of the Unit of Surgical Pathology of the Azienda Ospedaliero-Universitaria Pisana. For each patient, specimens (normal mucosa, early adenomas and carcinomas) were surgically obtained and fixed in 10% neutral-buffered formaldehyde and embedded in paraffin. Routine hematoxylin and eosin staining were performed on microtomic sections for histopathological examination. Histological diagnoses, reviewed independently by experienced pathologists (A.S., G.F.), were formulated according to the 2010 World Health Organization (WHO) Classification (4th Edition). Approval was granted by Azienda Ospedaliero-Universitaria Pisana Ethics Committee (protocol number 3867).

### Mutation analysis for *SMC1A*

DNA was extracted from embedded paraffin samples by the NucleoSpin Tissue kit (Macherey-Nagel) according to the manufacturer’s protocol. Primer pairs (Additional file 2: Table S3) were designed to amplify exons, exon/intron boundaries and short flanking intronic sequences. Amplified PCR products were purified and sequenced.

### Immunohistochemistry

SMC1A immunohistochemical analysis was performed on 3-μm tissue sections. After deparaffinization via serial xylene baths, samples were rehydrated via a graded ethanol series. Then the sections were heated to 98 °C for 40 min to unmask target antigens, cooled in solution at room temperature and washed with phosphate-buffered saline (PBS) for 3 min. After treatment with peroxide block, sections were washed again in PBS and incubated with power block reagent (Biogenex Laboratories) for 30 min. Primary mouse monoclonal antibody against human SMC1A (diluted 1:400, Bethyl Laboratories) was applied overnight at 4 °C. After washing with PBS, immunoreactivity was obtained by using the “Super Sensitive Polymer-HRP Detection System” (Biogenex), following the manufacturer’s instructions. Thereafter, samples were incubated for 20 min at room temperature with Super Enhancer Reagent, followed by incubation with Poly-HRP reagent. The reaction was developed using 0.05% 3,3′-diaminobenzidine tetrahydrochloride until adequate color development was seen. A negative control was obtained by substituting primary antibody with PBS. The staining percentage was graded as 0 (0–5%), 1 (6–20%), 2 (21–60%) and 3 (61–100%), and the staining intensity was graded as 0 (negative), 1 (+, weak), 2 (++, moderate) and 3 (+++, strong), as previously described [41, 42].

### *SMC1A* cDNA mutagenesis and cell transfection

Site-directed mutagenesis of the *SMC1A* cDNA (OriGene) was performed with QuikChange Site-Directed Mutagenesis Kit (Stratagene) according to the manufacturer’s instructions. Coding sequences for *SMC1A* wild type or mutated were inserted into the *Not* I site of the pcDNA3.1 plasmid (Invitrogen, ThermoFisher Scientific). The presence and orientation of the insert was confirmed by enzyme restriction digestion.

Human colorectal carcinoma HCT116 cells were maintained in culture according to American Type Culture Collection specifications. Briefly, cells were grown in McCoy’s Medium with 10% fetal calf serum and antibiotics in a humidified 5% CO_2_ atmosphere. HCT116 cells were transfected with the pcDNA3.1 vectors expressing *SMC1A* wild-type or *SMC1A* mutant. Stable expressing clones were obtained by selection in G418 (800 μg/ml) for 2 weeks.

### Animal care and experiments

Twenty-one immunocompromised NOD-SCID female mice 5–6 weeks of age were purchased from the Animal Care Facility of the IRCCS - Ospedale Policlinico San Martino of Genova, where animals were then housed and maintained in pathogen-free conditions. All experiments were reviewed and approved by the internal Review Board (OPBA) and authorized by the Italian Ministry of Health, accordingly with the current national and European regulations and guidelines for the care and use of laboratory animals (D.L. 26/2014; 86/609/EEC Directive).

Mice were bilaterally injected subcutaneously with 2 × 10^6^ HCT116 cells, HCT116 overexpressing SMC1A wild-type or HCT116 harboring *SMC1A* c.A2027G mutation, suspended in 0.1 ml of PBS. The mice were then regularly palpated to assess tumor latency. Tumor growth was recorded measuring nodule size with calipers three times per week. When the first nodule reached the volume of 300 mm^3^, all animals were sacrificed and tumors were excised, measured, photographed, weighed and formalin-fixed and snap-frozen in liquid nitrogen for further analyses.

For histology analysis, half of the samples were fixed in 4% neutral-buffered formalin, embedded in paraffin and cut to obtain 3- to 4-mm-thick sections. Slides were then stained with hematoxylin and eosin for histopathological examination; the other halves were snap-frozen in liquid nitrogen and kept at − 80 °C for molecular investigation.

### Library preparation and RNA-sequencing (RNA-seq)

Three tumors deriving from the inoculation of HCT116 (907_1, 907_2 and 907_3), four deriving from HCT116 overexpressing *SMC1A* wild-type (907_4, 907_5, 907_6 and 907_7) and four deriving from HCT116 harboring *SMC1A* c.A2027G mutation (907_8, 907_9, 907_10 and 907_11) were separately processed for RNA-seq analyses.

Library preparation was obtained using the TruSeq Stranded mRNA Sample Prep kit (Illumina). The poly-A mRNA was fragmented for 3 min at 94 °C and every purification step was performed using 1X Agencourt AMPure XP beads. Both RNA samples and final libraries were quantified using the Qubit 2.0 Fluorometer (Invitrogen) and the quality was tested using the Agilent 2100 Bioanalyzer RNA Nano assay (Agilent). Libraries were then processed with Illumina cBot for cluster generation on the flowcell, following the manufacturer’s instructions and sequenced on single-end mode on HiSeq 2500 (Illumina). The CASAVA 1.8.2 version of the Illumina pipeline was used to process raw data for format conversion and de-multiplexing.

### RNA-Seq analysis

To avoid low-quality data, adapters were removed by Cutadapt 1 and lower quality bases were trimmed by ERNE2. For the analysis of differentially expressed genes, the quality-checked reads were processed using the TopHat version 2.0.0 package (Bowtie 2 version 2.2.0) as FASTQ files [43–45]. Reads were mapped to the human reference genome GRCh37/hg19. Read abundance was evaluated and normalized by using Cufflinks 3 for each gene and Cuffdiff from the Cufflinks 2.2.0 package was used to calculate the differential expression levels and evaluate the statistical significance of detected alterations. Numbers of reads per sample are shown in Additional file 3: Table S4. Only protein-coding genes were considered and gene level expression values were determined by fragments per kilobase million (FPKM) mapped. All genes with FPKM > 1 were designated as expressed and analyzed with an established *p*-value < 0.05.

### cDNA synthesis and quantitative real-time PCR (qRT-PCR)

Total RNA was extracted by RNAeasy Mini-kit (Qiagen) and cDNA was synthesized with SuperScript™ II reverse transcriptase using oligo-dT (Invitrogen). PCR analyses were performed using Rotor Gene 3000 (Corbett). qPCR reactions were run in duplicate and normalized with respect to HPRT. Primers used for mRNA expression analysis are listed in Additional file 4: Table S5.

### Pathway analysis and function

The differentially expressed genes were functionally analyzed for biological processes using Database for Annotation, Visualization and Integrated Discovery (DAVID) v6.8 (https://david.ncifcrf.gov). For each term, the *p*-value was calculated and a term with *p* < 0.05 was considered to be enriched.

### Statistical analysis

LOH, CNVs and genome changed data were analyzed by Kruskal-Wallis test, a non-parametric method for comparing two or more independent samples of equal or different sample sizes. Immunohistochemistry data were analyzed by the Wilcoxon signed-rank test, a non-parametric method used for comparing related samples or matched samples. All other data were analyzed by Student’s *t*-test or the chi-squared test. *P*-values of *<* 0.05 were considered statistically significant.

### Accession number

The data discussed in this publication have been deposited in NCBI’s Gene Expression Omnibus and are accessible through GEO Series accession number GSE113678.

## Results

### OncoScan analysis during CRC development highlights the presence of extra-copies of cohesin genes

**Fig. 1:**
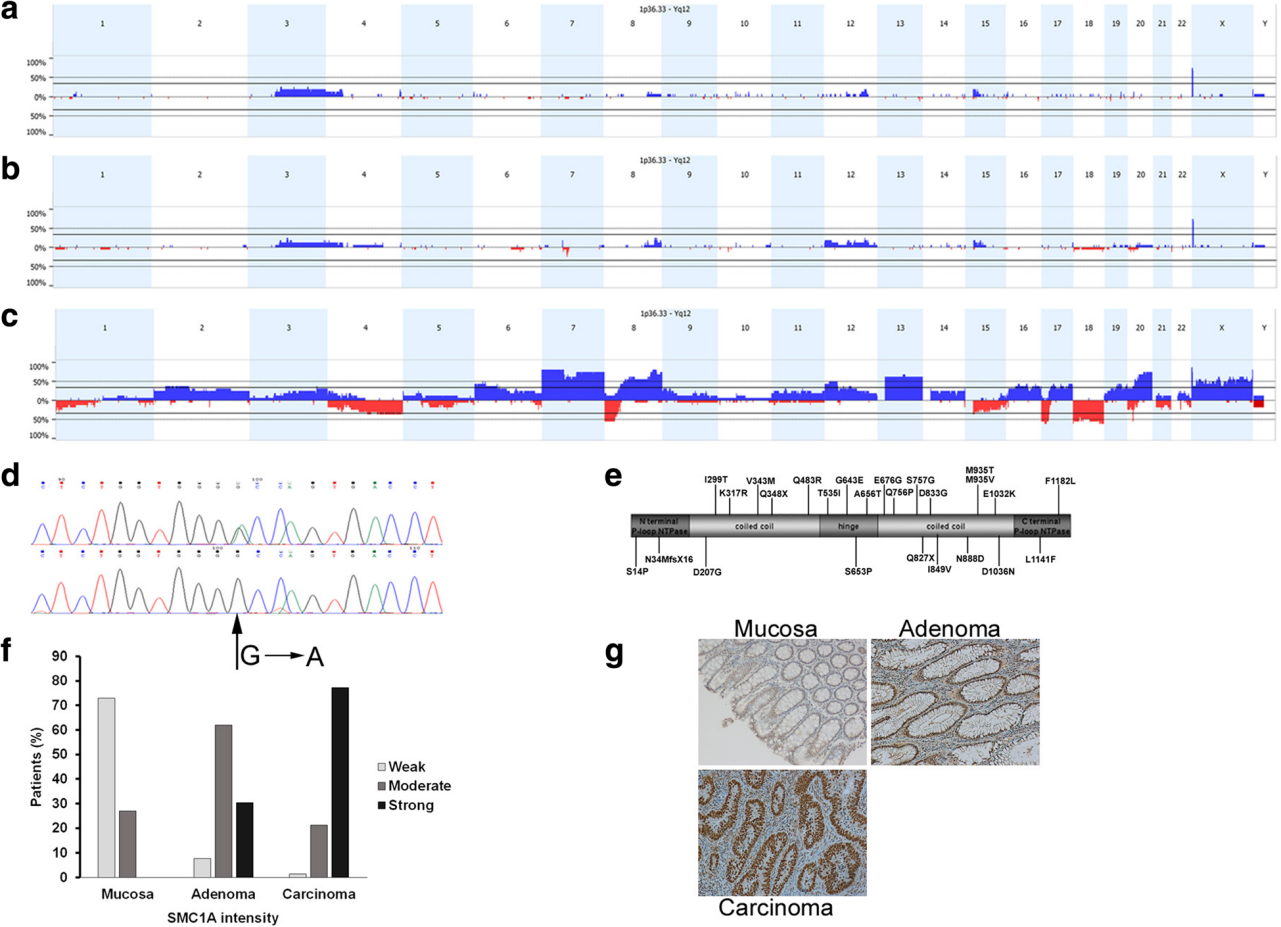
Comprensive analysis of *SMC1A*-cohesin gene at different steps during CRC development. OncoScan was used to obtain both CNVs and LOH in mucosa (*n* = 16), early adenoma (*n* = 16) and carcinoma (*n* = 16) samples. **a** CNVs profile in mucosa. **b** CNVs profile in adenomas. **c** CNVs profile in carcinoma showing the gain of whole chromosomes 7, 13, and X and the loss of chromosome 18. **d** Mutational screening in colorectal early adenomas and carcinoma allowed us to identify twenty-five *SMC1A* mutations (16 mutations in carcinomas and 9 in adenomas, see Table 2). Example of representative *SMC1A* sequencing is reported showing the nucleotide change c.G1966A (leading to p.A656T amino acid change) identified in patient 6. **e** Diagram of the SMC1A protein with mutations identified in carcinomas (above) and adenomas (below). The protein length is not in scale. **f** Percentage of subjects (*n* = 66) analyzed by SMC1A immunohistochemistry and showing strong, moderate and weak intensity. **g** Examples of representative immunohistochemistry results are shown. It is evident that the expression of SMC1A protein increased during cancer development

In order to identify copy number variations (CNVs) and loss of heterozygosity (LOH), we performed molecular cytogenetic analysis on normal mucosa, early adenoma, and carcinoma from 16 subjects affected by CRC. Genomic DNA was interrogated using OncoScan FFPE Array. We found an increase in LOH at the carcinoma stage (*p* = 0.0015, Kruskal-Wallis test, Table 1, Additional file 5: Figure S1a). In addition, most of the subjects (13/16) showed a marked increase in chromosome gains or losses proceeding from normal mucosa until the carcinoma stage (*p* = 0.0006, Fig. 1a-c, Additional file 5: Figures S1b–S4,). Common events in carcinoma were the gain of whole chromosomes 7, 13, and X; gain of the long arm of chromosomes 8 and 20; loss of chromosome 18 and loss of the short arm of chromosomes 8 and 17 (Fig. 1c, Additional file 5: Figure S4). Of note, few events were identified in both normal mucosa and early adenomas (Fig. 1a-b, Additional files 5: Figures S2 and S3). The distribution of the events on chromosomes during cancer development is shown in Additional file 5: Figure S5a, b and c. Globally, the fraction of the genome that was altered ranged from 8.99% in normal mucosa to 68.9% in carcinoma (*p* < 0.0001, Table 1, Additional file 5: Figure S1c).

**Table 1:** CNVs, LOH and genome changed during CRC development

Sample	CNV events	LOH (%)	Genome changed (%)
Mucosa	1–47	0–29.8	0.0058–8.99
Adenoma	3–71	0.1–24.1	0.0054–15.6
Carcinoma	1–156	0.3–34.9	0.012–68.9

Next, we manually searched for genes belonging to the cohesin pathway. This allowed us to identify genomic regions containing *HDAC8* (chromosome X, gain in 62.5% of subjects), *RAD21* (chromosome 8, gain in 75% of subjects), *STAG2* (chromosome X, gain in 62.5% of subjects) and *SMC1A* (chromosome X, gain in 50% of subjects). This finding indicates that cohesin genes go through extra-copies during CRC development.

### *SMC1A* sequencing reveals high frequency of mutations in carcinoma during CRC development

Next, we focused on *SMC1A* gene because its mutations have been identified in CRC and in other cancers and cause CIN and chromosome aneuploidy in vitro [29, 30]. In addition, accumulating evidence has proved that *SMC1A* plays a role in genome stability. In fact, it is involved in G2/M checkpoint and it is phosphorylated by both ATR and ATM protein kinase [12, 13, 46–48]. Sequencing of *SMC1A* revealed mutations in 13 out of 16 samples previously analyzed by OncoScan. We identified five synonymous variants (Additional file 6: Table S6) in carcinoma samples alone and twenty-five mutations: twenty-two missense mutations, a 1-bp deletion causing a frameshift and two non-sense mutations (Table 2, Fig. 1d). It is worth noting that 5 out of 13 carcinoma samples carried from two to four *SMC1A* mutations (Table 2, Additional file 7: Figure S6) whereas two subjects (9 and 12) carried the same mutation (c.A950G) at the carcinoma stage (Table 2). Amino acid changes encompass all gene domains without significant differences (Fig. 1e). The effect of mutations has been predicted in silico using the Mutation Taster program (http://www.mutationtaster.org/) and PolyPhen2 (http://genetics.bwh.harvard.edu/pph2/). Most missense mutations identified in carcinoma were predicted to be damaging (have a functional effect) while only two (with MutationTaster) or three (with PolyPhen2) mutations in adenomas (Additional file 8: Table S7) were damaging.

**Table 2:** *SMC1A* mutations identified in both adenoma and carcinoma samples

Subject	Gender	Stage	Exon	Nucleotide change	Amino acid change
1	M	Carcinoma	6	c.C1042T	p.Q348X
		Adenoma			
		Mucosa			
2	M	Carcinoma	14	c.A2267C	p.Q756P
			14	c.A2269G	p.S757G
			20	c.G3094A	p.E1032K
			24	c.T3544C	p.F1182 L
		Adenoma	16	c.2479 T	p.Q827X
		Mucosa			
3	M	Carcinoma	9	c.A1448G	p.Q483R
		Adenoma	1	c.T40C	p.S14P
		Mucosa			
4	M	Carcinoma	18	c.A2803G	p.M935 V
			18	c.T2804C	p.M935 T
			16	c.A2498G	p.D833G
		Adenoma			
		Mucosa			
5	F	Carcinoma			
		Adenoma	1	c.101delA	p.N34MfsX16
		Mucosa			
6	M	Carcinoma	12	c.G1928A	p.G643E
			12	c.G1966A	p.A656T
		Adenoma	17	c.A2662G	p.N888D
		Mucosa			
7	M	Carcinoma	10	c.C1604T	p.T535I
		Adenoma			
		Mucosa			
8	M	Carcinoma			
		Adenoma	4	c.A620G	p.D207G
		Mucosa			
9	F	Carcinoma	6	c.T896C	p.I299T
			6	c.A950G	p.K317R
		Adenoma	22	c.C3421T	p.L1141F
		Mucosa			
10	M	Carcinoma			
		Adenoma	16	c.A2545G	p.I849V
		Mucosa			
11	F	Carcinoma			
		Adenoma	20	c.G3106A	p.D1036N
		Mucosa			
12	M	Carcinoma	6	c.A950G	p.K317R
			6	c.G1027A	p.V343 M
		Adenoma	12	c.T1957C	p.S653P
		Mucosa			
13	M	Carcinoma	13	c.A2027G	p.E676G
		Adenoma			
		Mucosa			
14	F	Carcinoma			
		Adenoma			
		Mucosa			
15	M	Carcinoma			
		Adenoma			
		Mucosa			
16	M	Carcinoma			
		Adenoma			
		Mucosa			

### SMC1A expression increases from normal mucosa to carcinoma during CRC development

To investigate the levels of SMC1A expression during CRC development, we measured its expression in 66 subjects (including those analyzed by OncoScan assay) by immunochemistry, and again for each patient we analyzed normal mucosa, early adenoma and carcinoma. Robust SMC1A expression was observed in most of the carcinomas, 77.27% with strong (+++), 21.21% with moderate (++) and 1.52% with weak intensity (+). In contrast, only 30.3% of adenomas had strong intensity, whereas a significant fraction (62%) showed moderate intensity (Fig. 1f, Table 3, Additional file 9: Figures S7, S8). According to the Wilcoxon signed-rank test, the increase in SMC1A expression from normal mucosa to adenoma (*p* = 1.44 e-12) and from adenoma to carcinoma (*p* = 3.82 e-07) was highly significant. Immunohistochemistry data was validated by quantitative RT-PCR (Additional file 9: Figure S9). In normal mucosa, cells displayed a round shape, and since SMC1A is a nuclear protein, the signal was confined to the nuclei which were in the periphery. During malignant transformation, we observed that nuclei increased in size and occupied most of the cytoplasm (Fig. 1g, Additional file 10: Figure S10). It is likely that this phenomenon is caused by the chromosome aneuploidies we detected by OncoScan assay. Altogether, this data indicates that the expression of SMC1A increases during colorectal tumorigenesis, from normal mucosa to carcinoma.

**Table 3:** Analysis of SMC1A expression in normal mucosa, early adenoma and carcinoma samples

Subject	Stage	SMC1A (positive cells, %)	SMC1A (staining intensity)
1	Carcinoma	90%	+++
	Adenoma	90%	++
	Mucosa	80%	+
2	Carcinoma	90%	++
	Adenoma	90%	++
	Mucosa	80%	+
3	Carcinoma	90%	+++
	Adenoma	90%	+++
	Mucosa	90%	++
4	Carcinoma	90%	++
	Adenoma	60%	+
	Mucosa	60%	+
5	Carcinoma	90%	+++
	Adenoma	80%	+
	Mucosa	80%	+
6	Carcinoma	90%	++
	Adenoma	90%	++
	Mucosa	90%	+
7	Carcinoma	90%	+++
	Adenoma	70%	++
	Mucosa	80%	++
8	Carcinoma	90%	+
	Adenoma	70%	++
	Mucosa	70%	++
9	Carcinoma	80%	++
	Adenoma	70%	++
	Mucosa	80%	+
10	Carcinoma	90%	+++
	Adenoma	90%	+++
	Mucosa	90%	+
11	Carcinoma	90%	+++
	Adenoma	90%	+++
	Mucosa	80%	++
12	Carcinoma	90%	+++
	Adenoma	80%	+
	Mucosa	80%	+
13	Carcinoma	90%	++
	Adenoma	70%	++
	Mucosa	80%	+
14	Carcinoma	70%	+++
	Adenoma	80%	+++
	Mucosa	80%	+
15	Carcinoma	90%	+++
	Adenoma	90%	++
	Mucosa	90%	+
16	Carcinoma	90%	+++
	Adenoma	90%	+++
	Mucosa	70%	++
17	Carcinoma	80%	+++
	Adenoma	90%	+
	Mucosa	60%	+
18	Carcinoma	80%	++
	Adenoma	90%	++
	Mucosa	80%	+
19	Carcinoma	90%	+++
	Adenoma	70%	++
	Mucosa	80%	+
20	Carcinoma	90%	++
	Adenoma	90%	++
	Mucosa	90%	+
21	Carcinoma	80%	+++
	Adenoma	90%	+++
	Mucosa	40%	+
22	Carcinoma	90%	++
	Adenoma	70%	++
	Mucosa	40%	+
23	Carcinoma	20%	++
	Adenoma	80%	++
	Mucosa	60%	+
24	Carcinoma	90%	+++
	Adenoma	90%	+++
	Mucosa	90%	++
25	Carcinoma	90%	+++
	Adenoma	70%	++
	Mucosa	70%	+
26	Carcinoma	90%	+++
	Adenoma	80%	+++
	Mucosa	90%	++
27	Carcinoma	90%	++
	Adenoma	80%	++
	Mucosa	80%	++
28	Carcinoma	80%	+++
	Adenoma	90%	+++
	Mucosa	90%	++
29	Carcinoma	90%	+++
	Adenoma	80%	++
	Mucosa	80%	+
30	Carcinoma	80%	+++
	Adenoma	70%	++
	Mucosa	90%	++
31	Carcinoma	80%	++
	Adenoma	90%	++
	Mucosa	70%	++
32	Carcinoma	80%	+++
	Adenoma	80%	++
	Mucosa	10%	+
33	Carcinoma	90%	+++
	Adenoma	80%	++
	Mucosa	60%	+
34	Carcinoma	90%	+++
	Adenoma	90%	++
	Mucosa	70%	+
35	Carcinoma	90%	+++
	Adenoma	70%	+++
	Mucosa	80%	+
36	Carcinoma	90%	+++
	Adenoma	90%	++
	Mucosa	90%	+
37	Carcinoma	90%	+++
	Adenoma	70%	+
	Mucosa	80%	+
38	Carcinoma	90%	+++
	Adenoma	90%	+++
	Mucosa	90%	+
39	Carcinoma	90%	+++
	Adenoma	80%	+++
	Mucosa	90%	+
40	Carcinoma	90%	+++
	Adenoma	90%	+++
	Mucosa	80%	+
41	Carcinoma	90%	+++
	Adenoma	90%	++
	Mucosa	70%	+
42	Carcinoma	20%	++
	Adenoma	80%	++
	Mucosa	80%	+
43	Carcinoma	90%	+++
	Adenoma	60%	++
	Mucosa	90%	++
44	Carcinoma	90%	++
	Adenoma	90%	+++
	Mucosa	60%	+
45	Carcinoma	80%	+++
	Adenoma	60%	++
	Mucosa	80%	++
46	Carcinoma	90%	+++
	Adenoma	90%	+++
	Mucosa	80%	+
47	Carcinoma	90%	+++
	Adenoma	80%	++
	Mucosa	90%	++
48	Carcinoma	90%	+++
	Adenoma	90%	+++
	Mucosa	90%	+
49	Carcinoma	90%	+++
	Adenoma	90%	++
	Mucosa	80%	++
50	Carcinoma	90%	+++
	Adenoma	80%	++
	Mucosa	80%	+
51	Carcinoma	90%	+++
	Adenoma	70%	++
	normale	80%	++
52	Carcinoma	80%	++
	Adenoma	80%	++
	Mucosa	70%	+
53	Carcinoma	90%	+++
	Adenoma	80%	++
	Mucosa	90%	+
54	Carcinoma	90%	+++
	Adenoma	80%	+++
	Mucosa	90%	++
55	Carcinoma	80%	+++
	Adenoma	90%	++
	Mucosa	80%	+
56	Carcinoma	90%	+++
	Adenoma	80%	++
	Mucosa	90%	++
57	Carcinoma	90%	+++
	Adenoma	90%	++
	Mucosa	90%	+
58	Carcinoma	90%	+++
	Adenoma	90%	++
	Mucosa	80%	+
59	Carcinoma	70%	+++
	Adenoma	70%	++
	Mucosa	80%	+
60	Carcinoma	90%	+++
	Adenoma	80%	+++
	Mucosa	80%	+
61	Carcinoma	90%	+++
	Adenoma	90%	++
	Mucosa	90%	++
62	Carcinoma	90%	+++
	Adenoma	90%	++
	Mucosa	90%	+
63	Carcinoma	90%	+++
	Adenoma	80%	++
	Mucosa	80%	+
64	Carcinoma	80%	+++
	Adenoma	90%	+++
	Mucosa	90%	+
65	Carcinoma	90%	+++
	Adenoma	90%	+++
	Mucosa	90%	+
66	Carcinoma	80%	+++
	Adenoma	60%	++
	Mucosa	90%	+

### *SMC1A* overexpression reduces the latency period of cancer formation in vivo

**Fig. 2:**
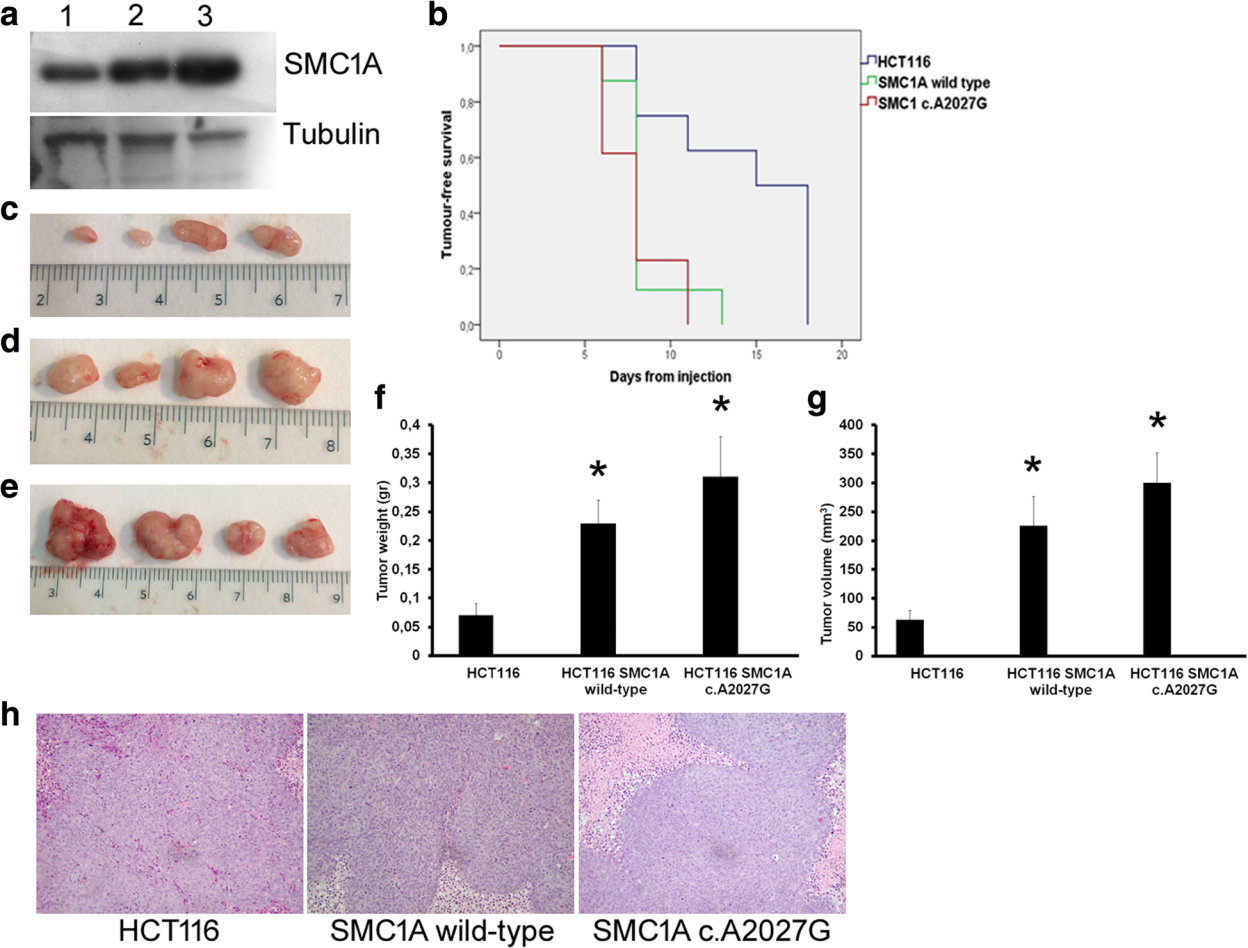
Effects of *SMC1A* mutation and overexpression in vivo. **a** HCT116 cells, a colorectal cancer cell line with stable karyotype, were stably transfected with both *SMC1A* wild-type gene and *SMC1A* c.A2027G mutation. Western blot shows a more marked expression in HCT116 *SMC1A* wild-type (2) and HCT116 *SMC1A* c.A2027G (3) when compared with HCT116 cells (1). An antibody against Tubulin was used as loading control. **b** The effects of HCT116, HCT116 *SMC1A* wild-type and HCT116 *SMC1A* c.A2027G cells were analyzed in vivo using an immunocompromised mouse model. Time-dependent analysis shows that HCT116 *SMC1A* wild-type and HCT116 *SMC1A* c.A2027G require 11 and 13 days to form tumors. In contrast, the development of tumors peaked after 18 days with HCT116 cells. **c** Representative images of tumors formed in the mice with HCT116 cells. **d** Tumors induced by HCT116 *SMC1A* wild-type. **e** Tumors formed in the mice in which HCT116 *SMC1A* c.A2027G cells were implanted. **f** Change in tumor weight. **g** Difference in tumor volume after subcutaneous cell inoculation. **h** Example of representative histopathological examination performed with hematoxylin and eosin staining. Enlargement 500x. *p < 0.05

To determine the effects of altered cohesin expression in vivo, we introduced both *SMC1A* wild-type gene and *SMC1A* c.A2027G (leading to p.E676G change, located in the coiled-coil domain near the hinge domain) mutation, which induced chromosomal instability when transiently transfected in vitro [30], in HCT116 cells, a near-diploid human colorectal cancer cell line with stable karyotype. Western blots were then performed to document that the vectors led to overexpression of both wild-type and mutated SMC1A proteins (Fig. 2a). The effect of *SMC1A* overexpression was analyzed in vivo using immunocompromised mouse models. The time-dependent analysis showed that the development of tumors peaked after 11 and 13 days (expressed as 50% of survival) with *SMC1A* c.A2027G mutation and *SMC1A* wild-type respectively, whereas the latency was longer (18 days) with HCT116 cells (*p* < 0.05, Fig. 2b). Furthermore, both the weight and volume of the tumors significantly increased in mice inoculated with *SMC1A* wild-type (*p* = 0.011, *p* = 0.0079) and *SMC1A* c.A2027G (*p* = 0.013, *p* = 0.0022) when compared with HCT116 cells (Fig. 2c-g, Additional file 11: Figure S11). No difference was found between *SMC1A* wild-type and *SMC1A* c.A2027G, though both the weight and volume of the latter were higher (Fig. 2f-g). Tumors derived from the three cell lines were morphologically indistinguishable from one another, and presented large necrotic areas and several mitotic figures (Fig. 2h).

Next, we measured global gene expression profiles in eleven tumors induced by parental and transduced HCT116 cells. In particular, three tumors induced by parental cells (907_1, 907_2 and 907_3), four induced by *SMC1A* wild-type transduced cells (907_4, 907_5, 907_6 and 907_7) and four induced by *SMC1A* c.A2027G transduced cells (907_8, 907_9, 907_10 and 907_11). Unsupervised sample clustering by principal component analysis (PCA) showed that tumors induced by mutant-*SMC1A* transduced cells differed to a greater extent than those induced by parental and wild-type *SMC1A* transduced cells (Additional file 12: Figure S12).

**Fig. 3:**
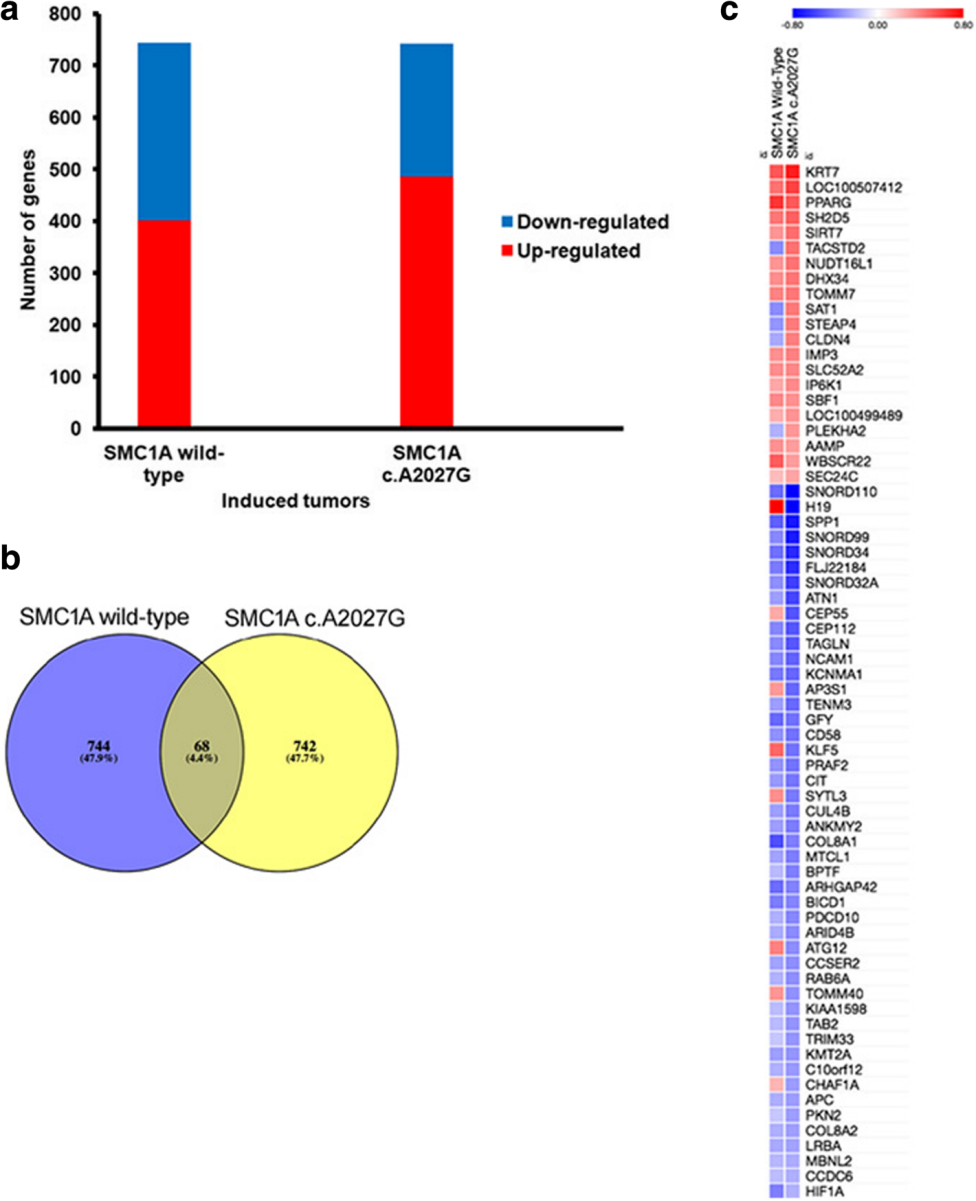
Gene expression profile in induced tumors. **a** RNA-seq data shows that HCT116 *SMC1A* wild-type induced tumors display 401 up- and 343 down-regulated genes, whereas HCT116 *SMC1A* c.A2027G-induced tumors show 486 up- and 256 down-regulated genes. **b** Venn diagram showing that HCT116 *SMC1A* wild-type and HCT116 *SMC1A* c.A2027G share sixty-eight dysregulated genes. **c** Heatmap of sixty-eight dysregulated genes showing that the transcriptional effects were very small, with fold changes ranging from + 0.8 to − 0.8

*SMC1A* wild-type and *SMC1A* c.A2027G displayed 744 (401 down and 343 up) and 742 (486 down and 256 up) dysregulated genes respectively when compared with parental HCT116-induced tumors (Fig. 3a). The transcriptional effects were small, with fold changes ranging from + 0.9 to − 0.17 and from + 0.79 to − 1.00 for *SMC1A* wild-type and *SMC1A* c.A2027G respectively (Additional file 12: Tables S8, S9). Dysregulated genes belong to many biological processes (Additional file 13: Figures S13, S14, Tables S10, S11). Furthermore, 68 dysregulated genes were shared in common between tumors induced by *SMC1A* wild-type and *SMC1A* c.A2027G (Fig. 3b). All of them displayed only minor fold changes that ranged from + 0.8 to − 0.8 when compared with the baseline of parental HCT116 (Fig. 3c). RNA-seq data was validated in eleven genes (*ATG12*, *CEP55*, *CHAF1A*, *CLDN4*, *H19*, *HIF1A*, *KLF5*, *SAT1*, *STEAP4*, *TACSTD2* and *TOMM40*) by quantitative RT-PCR experiments (Additional file 14, Figures S15, S16). These genes were chosen because they showed differential expression between *SMC1A* wild-type and *SMC1A* c.A2027G tumors (Fig. 3c). In addition, since most of them are involved in cancer development their differential expression could explain the aggressiveness of SMC*1A* c.A2027G tumors.

## Discussion

CRC is the third most common human malignancy worldwide, with an estimated 135,430 new cases and 50,260 deaths in 2017 in the United States alone [49]. CRC progresses through a series of histopathologic stages ranging from dysplastic crypts to malignant cancers. Most CRC is characterized by CIN, and clinical management of these tumors poses a serious dilemma due to its worse prognosis. Though the molecular basis for CIN is just beginning to be discovered, it has been suggested that CIN is an early event in cancer development, initiating CRC.

To characterize somatic alteration in CRC, 48 samples (16 normal mucosa, 16 adenomas and 16 carcinomas) were profiled for CNVs with OncoScan. We found the gain of chromosomes 7, 13, X, 8q and 20q and the loss of chromosomes 18, 8p and 17p in CRC samples. Interestingly, these chromosome changes were previously described in molecular characterization of CRC reports [50, 51]. However, we did not detect the loss of chromosomes 14q and 15q. In addition, we found that RAD21 was amplified in 75% of patients against only 2% in The Cancer Genome Atlas (TCGA) data of CRC [51]. It is likely that these discrepancies are due to the number of samples analyzed (48 in this work vs 276 in [51]).

*SMC1A* gene has been postulated to participate in CRC development by promoting aneuploidy [29, 30] but the biological basis of cohesin involvement is currently unknown. Here we report that colorectal tissue acquires extra-copies of *SMC1A* gene during tumorigenesis. In addition, SMC1A expression is significantly more robust in carcinomas than in normal mucosa and early adenomas. Recently, the overexpression of SMC1A was identified as a predictor of poor prognosis in late-stage CRC [42]. These findings suggest that overexpression of SMC1A plays a role in cancer pathogenesis. This notion is corroborated by the finding that human primary fibroblasts overexpressing SMC3, the molecular partner of SMC1A in the cohesin core, showed evidence of cell transformation, including anchorage-independent growth and foci of transformation [52].

Interestingly, in addition to SMC1A overexpression, carcinoma samples display more *SMC1A* mutations than adenomas and about 40% of carcinomas are characterized by carrying several *SMC1A* mutations in the same sample, suggesting that different clonal populations arise during cancer development. Furthermore, the finding that neutral mutations were identified only in carcinomas suggests that the mutation rate of SMC1A is higher in carcinoma than in the early stage of cancer development. Previously we showed that SMC1A mutations decrease from early adenomas to colorectal cancers [30]. This apparent contradiction could be due to sample selection. In fact, in the present work we selected only patients whose mucosa, adenoma and carcinoma were available while in a previous work [30] adenoma and carcinoma samples were selected independently. We postulate that the combination of both *SMC1A* mutations and gene expression up-regulation may contribute to generating the additional genetic lesions required for a cell to fully undergo malignant transformation.

The ectopic expression of *SMC1A* has a positive impact on in vivo growth. In fact, the overexpression of *SMC1A* reduced the latency period of cancer formation, and both the size and volume of tumors in a subcutaneous murine xenograft model were significantly increased in presence of up-regulated *SMC1A*. Of note, tumors induced by *SMC1A* c.A2027G mutation were bigger than those induced by *SMC1A* overexpression alone, indicating that mutation has an additive positive effect on tumor growth.

SMC1A up-regulation resulted in a significant change in gene expression profiles. In fact, we found more than 700 dysregulated genes with small fold changes ranging from + 0.9 to − 1.00. Automated analysis showed that differentially expressed genes were virtually implicated in many metabolic pathways, arguing a role for cohesin in cellular proliferation, signalling pathways and other transformation-associated processes. We identified a subset of genes that which showed differential expression between *SMC1A* wild-type and *SMC1A* c.A2027G tumors. *ATG12* and *STEAP4* genes are particularly interesting. ATG12 regulates the apoptotic pathway by binding and inactivating BCL2 and MCL1 [53] whereas STEAP4 is a metalloreductase, involved in responses to nutrients, inflammatory and oxidative stress, fatty acid metabolism, and glucose metabolism [54–56]. Recently, it has been found that *ATG12* and *STEAP4* were down- and up-regulated respectively in human CRC and predicted poor prognosis [57, 58]. Again, the dysregulation of *CEP55*, *CLDN4*, *CHAF1A*, *H19* and *STEAP4* have been found to significantly correlate with CRC tumor stage, aggressiveness, metastasis and poor prognosis [59–63]. Altogther, their differential expression could explain the greater aggressiveness of *SMC1A* c.A2027G tumors.

Furthermore, this data indicates that changes in *SMC1A* expression level have significant, though modest, effects on transcription throughout the genome and that tumorigenesis likely arises from the collective effects of small changes in the expression of many genes. At present, we do not know how many observed gene expression changes are primary (i.e., due to direct transcriptional actions of overexpressed cohesin) and how many are downstream consequences of cohesin dysregulation.

## Conclusions

**Fig. 4:**
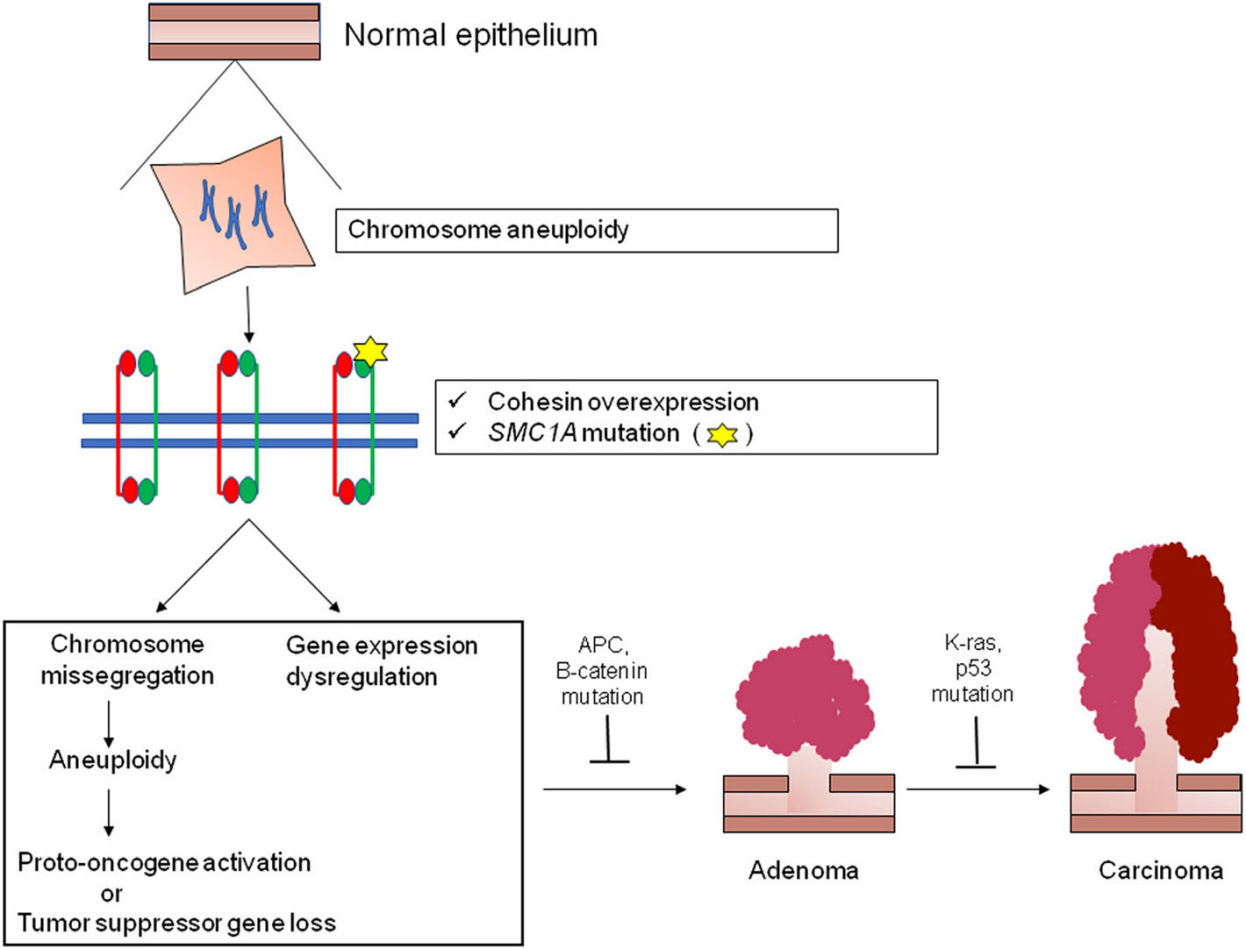
Cohesin and cancer. Abnormal cohesin activity leads to chromosome missegregation with chromosome loss and gain. These aneuploidies could alter the expression of proto-oncogenes or tumor suppressor genes. Furthermore, since cohesin binds the promoter regions of genes essential for cell cycle control, cohesin could affects directly the expression of tumor-promoting genes. These events could produce a genetic environment which favors additional changes with the acquisition of full malignant phenotype

In our model, early events of chromosome missegregation lead to the occurrence of extra-copies of X chromosome in normal mucosa (Fig. 4). As consequence, this causes the overexpression of *SMC1A* gene because it maps to Xp11.22–Xp11.21, in a region that escapes X inactivation. Cohesin ensures correct chromosome segregation and its overexpression may lead to the failure of this process, leading to chromosome imbalance, with abnormal numbers of chromosomes in daughter cells. Mitotic missegregation can contribute to cancerogenesis in two different ways. Chromosome gain results in the activation of proto-oncogenes. For instance, the trisomy of chromosome 7 is associated with the overexpression of *MET* oncogene in renal carcinoma [64]. Chromosome loss could lead to the removal of tumor suppressor gene resulting in tumorigenesis if the first allele is inactivated. The loss of chromosome 10 results in the inactivation of tumor suppressor gene *PTEN* in human glioblastoma [65].

In addition to its role in mediating sister chromatid cohesion, cohesin plays a pivotal role in gene transcription regulation. In fact, cohesin binds the chromatin near the transcription start site of many genes, including *c-MYC,* important for cell cycle regulation, differentiation and development [66–68]. Interestingly, genome-wide data has showed the presence of genomic sites where the co-localization of cohesin and c-MYC has a relevant role in co-regulating transcription [69]. These findings allow us to hypothesize that cohesin overexpression results in transcription changes modulating the expression of specific biochemical tumor-promoting pathways.

In addition to X chromosome aneuploidy, we found that *SMC1A* was also mutated. The presence of a mutated SMC1A subunit could produce an inactive or functionally restricted cohesin complex. We showed that *SMC1A* mutations affect the association of SMC hinge dimers with DNA [25], suggesting a potential role of SMC proteins in remodeling chromatin architecture. We propose that both cohesin overexpression and *SMC1A* mutations accelerate the acquisition of a mutator phenotype driving tumorigenesis by triggering additional genetic changes that allow a growth advantage and CRC development (Fig. 4). This notion is supported by the observation that colorectal cancer cells exhibit up to 100-fold higher rates of missegregation than normal cells [70].

Collectively, our findings strongly suggest that *SMC1A* functions as a “caretaker” oncogene in CRC. This discovery has important clinical applications because *SMC1A* could serve as a potential target for the development of new therapies in CRC.

## Additional files

Additional file 1: **Table S1.** Features of CRC patients analyzed by OncoScan. **Table S2.** Features of CRC patients analyzed by immunohistochemistry. (PDF 167 kb)

Additional file 2: **Table S3.** Nucleotide primers used for amplifying the *SMC1A* gene. (PDF 23 kb)

Additional file 3: **Table S4.** Number of reads in tumor samples analysed by RNA-seq. (PDF 12 kb)

Additional file 4: **Table S5.** Primers sequences used for validating RNA-seq data by RT-qPCR. (PDF 41 kb)

Additional file 5: **Figure S1.** (**a**) Violin plot showing LOH during cancer progression. (**b**) Violin plot showing CNVs in mucosa, adenoma and carcinoma samples. (**c**) Violin plot showing the percentage of genome changed during tumorigenesis. **Figure S2.** CNVs profile in colorectal mucosa. Example of representative CNVs in subject 12 is reported. **Figure S3.** CNVs profile in colorectal adenoma. Example of representative CNVs in subject 12 is reported. **Figure S4.** CNVs profile in colorectal carcinoma. Example of representative CNVs in subject 12 is reported. The OncoScan analysis identified the gain of whole chromosomes 7, 13 and X; the partial gain of chromosomes 8 and 20; the loss of chromosome 18 and the partial loss of chromosomes 1, 2, 5, 8 and 17. **Figure S5.** (**a**) Circos plot showing the distribution of the events in mucosa samples. (**b**) Circos plot showing the distribution of the events in adenoma samples. (**c**) Circos plot showing the distribution of the events in carcinoma samples. Colored dots represent the events. The black circle line is the baseline: the dots inside represent the deletions and the outside ones show the amplifications. (PDF 14770 kb)

Additional file 6: **Table S6.** *SMC1A* synonymous variants identified in carcinoma samples. (PDF 30 kb)

Additional file 7: **Figure S6.** *SMC1A* mutational screening. Example of representative *SMC1A* sequencing is reported, showing multiple nucleotide changes in the carcinoma deriving from subject 2. (PDF 64 kb)

Additional file 8: **Table S7.** Prediction of mutation effects on SMC1A protein using Mutation Tester and PolyPhen2. (PDF 17 kb)

Additional file 9: **Figure S7.** Violin plot showing the distribution of SMC1A-positive cells. **Figure S8.** Violin plot showing the distribution of SMC1A staining intensity. **Figure S9.** Immunohistochemistry data was validated by RT-qPCR. *p < 0.05. (PDF 1153 kb)

Additional file 10: **Figure S10.** SMC1A immunohistochemistry in mucosa, adenoma and carcinoma (PDF 1996 kb)

Additional file 11: **Figure S11.** Effects of *SMC1A* mutation and overexpression in in vivo. (**a**) Representative images of tumours formed in the mice with HCT116 cells. (**b**) Tumours formed in the mice in which HCT116 *SMC1A* wild-type cells were implanted. (**c**) Tumours induced by HCT116 *SMC1A* c.A2027G cells. (PDF 1199 kb)

Additional file 12: **Figure S12.** Classification of three tumors deriving from the inoculation of HCT116 (907_1, 907_2 and 907_3, red circle), four deriving from HCT116 overexpressing *SMC1A* wild-type (907_4, 907_5, 907_6 and 907_7, blue circle) and four deriving from HCT116 harboring *SMC1A* c.A2027G mutation (907_8, 907_9, 907_10 and 907_11, green circle) by gene expression. **Table S8.** Dysregulated genes in HCT116 SMC1A wild-type induced tumors. **Table S9.** Dysregulated genes in HCT116 SMC1A c.A2027G induced tumors. (PDF 851 kb)

Additional file 13: **Figure S13.** GO term enrichment analysis of biological processes that were significantly overrepresented when considering differentially expressed genes in HCT116 *SMC1A* wild-type cells. **Figure S14.** GO term enrichment analysis of biological process that were significantly overrepresented when considering differentially expressed genes in HCT116 *SMC1A* c.A2027G cells. **Table S10.** Dysregulated pathways identified in tumors induced by *SMC1A* wild-type with *p* < 0.01. **Table S11.** Dysregulated pathways identified in tumors induced by *SMC1A* c.A2027G with p < 0.01. (PDF 3535 kb)

Additional file 14: **Figure S15.** Dysregulated genes in induced tumors. RNA-seq data was validated for eleven genes, *ATG12*, *CEP55*, *CHAF1A*, *CLDN4*, *H19*, *HIF1A*, *KLF5*, *SAT1*, *STEAP4*, *TACSTD2* and *TOMM40,* by RT-qPCR. *p < 0.05. **Figure S16.** Heatmap of the eleven dysregulated genes validated by RT-qPCR. (PDF 3904 kb)

## Abbreviations

AGCC: Affymetrix genechip command console
ATM: Ataxia-telangiectasia mutated
ATR: Ataxia-telangiectasia and Rad3 related
BAF: B-allele frequency
CIN: Chromosomal instability
CNV: Copy number variations
CRC: Colorectal cancer
FPKM: Fragments per kilobase million
HDAC8: Histone deacetylase 8
LOH: Loss of heterozygosity
MIP: Molecular inversion probe
NIPBL: Nipped-B homolog
PBS: Phosphate-buffered saline
PCA: Principal component analysis
qRT-PCR: Quantitative real-time PCR
SMC: Structural maintenance of chromosome
STAG: Stromal antigen
TCGA: The cancer genome atlas
