# Supplementary material for: Overexpression of the cohesin-core subunit SMC1A contributes to colorectal cancer development
Source: J Exp Clin Cancer Res. 2019 Mar 1;38:108. doi: 10.1186/s13046-019-1116-0 (PMC6397456; doi:10.1186/s13046-019-1116-0)
Supplement: Supplementary file 1 — Table S1. Features of CRC patients analyzed by OncoScan. Table S2. Features of CRC patients analyzed by immunohistochemistry. (PDF 167 kb) [file 13046_2019_1116_MOESM1_ESM.pdf]

Supplementary Table 1. Features of CRC patients analyzed by OncoScan.

Subject	Gender	Age (years)	Stage	TNM
**1**			Carcinoma	IV
	M	66	Adenoma	
			Mucosa	
**2**			Carcinoma	III
	M	83	Adenoma	
			Mucosa	
**3**			Carcinoma	III
	F	88	Adenoma	
			Mucosa	
**4**			Carcinoma	II
	M	80	Adenoma	
			Mucosa	
**5**			Carcinoma	III
	M	70	Adenoma	
			Mucosa	
**6**			Carcinoma	II
	F	60	Adenoma	
			Mucosa	
**7**			Carcinoma	I
	F	81	Adenoma	
			Mucosa	
**8**			Carcinoma	III
	M	76	Adenoma	
			Mucosa	
**9**			Carcinoma	III
	M	63	Adenoma	
			normale	
**10**			Carcinoma	III
	F	80	Adenoma	
			Mucosa	
**11**			Carcinoma	III
	M	83	Adenoma	
			Mucosa	
**12**			Carcinoma	III
	M	84	Adenoma	
			Mucosa	
**13**			Carcinoma	II
	M	69	Adenoma	
			Mucosa	
**14**			Carcinoma	III

	M	51	Adenoma	
			Mucosa	
**15**			Carcinoma	III
	M	78	Adenoma	
			Mucosa	
**16**			Carcinoma	II
	M	79	Adenoma	
			Mucosa	

Supplementary Table 2. Features of CRC patients analyzed by immunohistochemistry.

Subject	Gender	Age (years)	Stage	TNM
**1***			Carcinoma	IV
	M	66	Adenoma	
			Mucosa	
**2***			Carcinoma	III
	M	83	Adenoma	
			Mucosa	
**3***			Carcinoma	III
	F	88	Adenoma	
			Mucosa	
**4***			Carcinoma	II
	M	80	Adenoma	
			Mucosa	
**5**			Carcinoma	III
	F	81	Adenoma	
			Mucosa	
**6**			Carcinoma	III
	M	65	Adenoma	
			Mucosa	
**7***			Carcinoma	III
	M	70	Adenoma	
			Mucosa	
**8***			Carcinoma	II
	F	60	Adenoma	
			Mucosa	
**9***			Carcinoma	I
	F	81	Adenoma	
			Mucosa	
**10***			Carcinoma	III
	M	76	Adenoma	
			Mucosa	
**11***			Carcinoma	III
	M	63	Adenoma	
			normale	
**12***			Carcinoma	III
	F	80	Adenoma	
			Mucosa	
**13**			Carcinoma	II
	M	71	Adenoma	
			Mucosa	
**14***			Carcinoma	III

	M	83	Adenoma	
			Mucosa	
**15**			Carcinoma	III
	F	55	Adenoma	
			Mucosa	
**16**			Carcinoma	IV
	M	73	Adenoma	
			Mucosa	
**17**			Carcinoma	III
	M	52	Adenoma	
			Mucosa	
**18**			Carcinoma	II
	M	74	Adenoma	
			Mucosa	
**19**			Carcinoma	III
	M	84	Adenoma	
			Mucosa	
**20**			Carcinoma	II
	M	73	Adenoma	
			Mucosa	
**21***			Carcinoma	III
	M	84	Adenoma	
			Mucosa	
**22***			Carcinoma	II
	M	69	Adenoma	
			Mucosa	
**23***			Carcinoma	III
	M	51	Adenoma	
			Mucosa	
**24***			Carcinoma	III
	M	78	Adenoma	
			Mucosa	
**25**			Carcinoma	III
	M	69	Adenoma	
			Mucosa	
**26**			Carcinoma	IV
	M	75	Adenoma	
			Mucosa	
**27**			Carcinoma	II
	M	50	Adenoma	
			Mucosa	
**28**			Carcinoma	III
	M	64	Adenoma	

			Mucosa	
**29**			Carcinoma	III
	F	78	Adenoma	
			Mucosa	
**30**			Carcinoma	III
	F	83	Adenoma	
			Mucosa	
**31**			Carcinoma	II
	M	85	Adenoma	
			Mucosa	
**32**			Carcinoma	IV
	F	84	Adenoma	
			Mucosa	
**33**			Carcinoma	III
	M	89	Adenoma	
			Mucosa	
**34**			Carcinoma	III
	M	63	Adenoma	
			Mucosa	
**35**			Carcinoma	IV
	M	81	Adenoma	
			Mucosa	
**36**			Carcinoma	III
	M	51	Adenoma	
			Mucosa	
**37**			Carcinoma	III
	F	50	Adenoma	
			Mucosa	
**38**			Carcinoma	III
	F	70	Adenoma	
			Mucosa	
**39**			Carcinoma	II
	F	52	Adenoma	
			Mucosa	
**40**			Carcinoma	III
	M	63	Adenoma	
			Mucosa	
**41**			Carcinoma	III
	F	40	Adenoma	
			Mucosa	
**42***			Carcinoma	II
	M	79	Adenoma	
			Mucosa	

**43**			Carcinoma	IV
	M	49	Adenoma	
			Mucosa	
**44**			Carcinoma	II
	M	63	Adenoma	
			Mucosa	
**45**			Carcinoma	III
	M	58	Adenoma	
			Mucosa	
**46**			Carcinoma	III
	M	42	Adenoma	
			Mucosa	
**47**			Carcinoma	III
	F	78	Adenoma	
			Mucosa	
**48**			Carcinoma	IV
	F	70	Adenoma	
			Mucosa	
**49**			Carcinoma	III
	M	58	Adenoma	
			Mucosa	
**50**			Carcinoma	III
	M	72	Adenoma	
			Mucosa	
**51**			Carcinoma	III
	M	64	Adenoma	
			Mucosa	
**52**			Carcinoma	II
	M	59	Adenoma	
			Mucosa	
**53**			Carcinoma	III
	M	76	Adenoma	
			Mucosa	
**54**			Carcinoma	IV
	M	65	Adenoma	
			Mucosa	
**55**			Carcinoma	III
	F	83	Adenoma	
			Mucosa	
**56**			Carcinoma	III
	M	65	Adenoma	
			Mucosa	
**57**			Carcinoma	III

	M	55	Adenoma	
			Mucosa	
**58**			Carcinoma	IV
	F	72	Adenoma	
			Mucosa	
**59**			Carcinoma	III
	M	62	Adenoma	
			Mucosa	
**60**			Carcinoma	III
	F	51	Adenoma	
			Mucosa	
**61**			Carcinoma	III
	M	76	Adenoma	
			Mucosa	
**62**			Carcinoma	III
	F	73	Adenoma	
			Mucosa	
**63**			Carcinoma	III
	M	73	Adenoma	
			Mucosa	
**64**			Carcinoma	III
	M	55	Adenoma	
			Mucosa	
**65**			Carcinoma	III
	M	67	Adenoma	
			Mucosa	
**66**			Carcinoma	III
	M	75	Adenoma	
			Mucosa	

*Also analyzed by OncoScan
