# Supplementary material for: Overexpression of the cohesin-core subunit SMC1A contributes to colorectal cancer development
Source: J Exp Clin Cancer Res. 2019 Mar 1;38:108. doi: 10.1186/s13046-019-1116-0 (PMC6397456; doi:10.1186/s13046-019-1116-0)
Supplement: Supplementary file 2 — Table S3. Nucleotide primers used for amplifying the SMC1A gene. (PDF 23 kb) [file 13046_2019_1116_MOESM2_ESM.pdf]

Supplementary Table 3. Nucleotide primers used for amplifying the *SMC1A* gene (NCBI Reference Sequence: NG_006988.2)

Primer	Forward primer	Reverse primer	Size(bp)	Exon
1	CGTATACGCAACATCAGTCC	CGCGACGTTTCAGGTTACAT	379	1
2	GAGTACTGAGTGATTAGGGG	TGAGATGGAGCAGACAGAGT	597	2/3
2A	GAGTACTGAGTGATTAGGGG	CTCCTTCCTGTCCCAATCAA	371	2
2B	GTACCTTTGCCCGTGTCATT	TGAGATGGAGCAGACAGAGT	324	3
3	AATGGGTAAGGTGAACTGGG	AAACAGCACGGCCTCTTGGT	653	4/ 5
4	CTCCTTTGGGTGAAAAGCCT	GGATTTGGGATGCTCAACCT	642	6/ 7
5	ATGGTCTTGGCGTAAAGCCT	GATTGGCAACCCTGTCCTTA	586	8/ 9
5A	ATGGTCTTGGCGTAAAGCCT	TTCATCAATACGCCGCTTGG	366	8
5B	ACCTACATGCCCCTCTTCAT	GATTGGCAACCCTGTCCTTA	397	9
6	ATTAGGATTCCTGAGCCAGC	AATCTCCAGTACTGAGCCTG	580	10/ 11
6A	ATTAGGATTCCTGAGCCAGC	TCAGTCAGTGGCAGAACACA	334	10
6B	TGTGTTCTGCCACTGACTGA	AATCTCCAGTACTGAGCCTG	265	11
7	CAGGCTCAGTACTGGAGATT	AACCTAGGCCAGGAATGTGT	508	12/ 13
8	CCAATGCAGTCAAGGTAGCT	GATGTCAAGCTAGAGGCTCA	456	14/ 15
9	CCTGGGTCTAGTTTCCCTTT	GACATTATCCTTCTGTCTGTCG	324	16
10	AACTGCCTAGTAGGAAGGGT	CCTTCCTGGTCACTTTCACT	562	17/ 18
10A	AACTGCCTAGTAGGAAGGGT	CACCTCCTTCTGTAAATGGG	358	17
10B	ATTCGTAAGAAACTCGGGGG	CCTTCCTGGTCACTTTCACT	351	18
11	GTCTGCTCCACTTGACTCTC	CCTCTCTGGACAACTAGGAA	222	19
12	CCACACTCAGTCAGTCATCT	TGGCATACCCTTAGCCTCTT	298	20
13	TGTTCCTGCTCTGGATTGTC	TCACCTTCGCAGATCTCTGT	600	21/22
13A	TGTTCCTGCTCTGGATTGTC	CTGAGACTGGATGGAGATAG	321	21
13B	CTATCTCCATCCAGTCTCAG	TCACCTTCGCAGATCTCTGT	298	22
14	TCAGGCAACTTTGAGACCTG	TGGCCATTCAGGACCTGATT	737	23/ 24
14A	TCAGGCAACTTTGAGACCTG	TCCCCAGTATTTTCCAGCCA	455	23
14B	TCATCCATGCTGTGGTAGTC	TGGCCATTCAGGACCTGATT	391	24
15	TTTGGGCAGGTATGTAGGGA	GGAAGGTTGGGAGTCAAATATC	288	25
