# Supplementary material for: Overexpression of the cohesin-core subunit SMC1A contributes to colorectal cancer development
Source: J Exp Clin Cancer Res. 2019 Mar 1;38:108. doi: 10.1186/s13046-019-1116-0 (PMC6397456; doi:10.1186/s13046-019-1116-0)
Supplement: Supplementary file 3 — Table S4. Number of reads in tumor samples analysed by RNA-seq. (PDF 12 kb) [file 13046_2019_1116_MOESM3_ESM.pdf]

Supplementary Table 4. Number of reads in tumor samples analysed by RNA-seq

Sample	Number of reads
**HCT116**	
907_1	31149558
907_2	30456467
907_3	36544210
**HCT116 SMC1A wt**	
907_4	28562037
907_5	40149769
907_6	34742567
907_7	27359101
**HCT116 *SMC1A* c.A2027G**	
907_8	26658634
907_9	22050651
907_10	36008327
907_11	30471152
