# Supplementary material for: Overexpression of the cohesin-core subunit SMC1A contributes to colorectal cancer development
Source: J Exp Clin Cancer Res. 2019 Mar 1;38:108. doi: 10.1186/s13046-019-1116-0 (PMC6397456; doi:10.1186/s13046-019-1116-0)
Supplement: Supplementary file 4 — Table S5. Primers sequences used for validating RNA-seq data by RT-qPCR. (PDF 41 kb) [file 13046_2019_1116_MOESM4_ESM.pdf]

Supplementary Table 5. Primers sequences used for validating RNA-seq data by RT-qPCR

**Gene**	**Forward primer sequence (5'-3')**	**Reverse primer sequence (5'-3')**
ATG12	CTCAGTCGCTACTTCCGCTC	TTCGTGTTCGCTCTACTGCC
CHAF1A	TCCCATCTCGCTGAAGAGGA	CATACGTCACCCCTGCTCTC
CEP55	CAATCTCTGCCCGCTCTGAT	CTCAAGGACTCGAATTTTCTCCA
CLDN4	GGCCGGCCTTATGGTGATAG	AGTAAGGCTTGTCTGTGCGG
HIF1A	GACAAGCCACCTGAGGAGAG	GTGGCAACTGATGAGCAAGC
H19	GAGCACCTTGGACATCTGGAG	GCCCTCGATCCCCTAAACCT
KLF5	CCTCTCTCCCTGCTCATAGGC	ACTGGTCTACGACTGAGGCA
SAT1	CTCCGGAAGGACACAGCATT	AAACATGCAACAACGCCACT
STEAP4	CGCCTCTCCCTCAGTTATGG	CACAAACACCTGCCGACTTG
TACSTD2	TCACCAACCGGAGAAAGTCG	AGGAAGCGTGACTCACTTGG
TOMM40	CTCTGACCTCTCCCCTAGCAG	TGAGACTGCCACTGTTGTCC
