# Supplementary material for: Overexpression of the cohesin-core subunit SMC1A contributes to colorectal cancer development
Source: J Exp Clin Cancer Res. 2019 Mar 1;38:108. doi: 10.1186/s13046-019-1116-0 (PMC6397456; doi:10.1186/s13046-019-1116-0)
Supplement: Supplementary file 5 — Figure S1. (a) Violin plot showing LOH during cancer progression. (b) Violin plot showing CNVs in mucosa, adenoma and carcinoma samples. (c) Violin plot showing the percentage of genome changed during tumorigenesis. Figure S2. CNVs profile in colorectal mucosa. Example of representative CNVs in subject 12 is reported. Figure S3. CNVs profile in colorectal adenoma. Example of representative CNVs in subject 12 is reported. Figure S4. CNVs profile in colorectal carcinoma. Example of representative CNVs in subject 12 is reported. The OncoScan analysis identified the gain of whole chromosomes 7, 13 and X; the partial gain of chromosomes 8 and 20; the loss of chromosome 18 and the partial loss of chromosomes 1, 2, 5, 8 and 17. Figure S5. (a) Circos plot showing the distribution of the events in mucosa samples. (b) Circos plot showing the distribution of the events in adenoma samples. (c) Circos plot showing the distribution of the events in carcinoma samples. Colored dots represent the events. The black circle line is the baseline: the dots inside represent the deletions and the outside ones show the amplifications. (PDF 14770 kb) [file 13046_2019_1116_MOESM5_ESM.pdf]

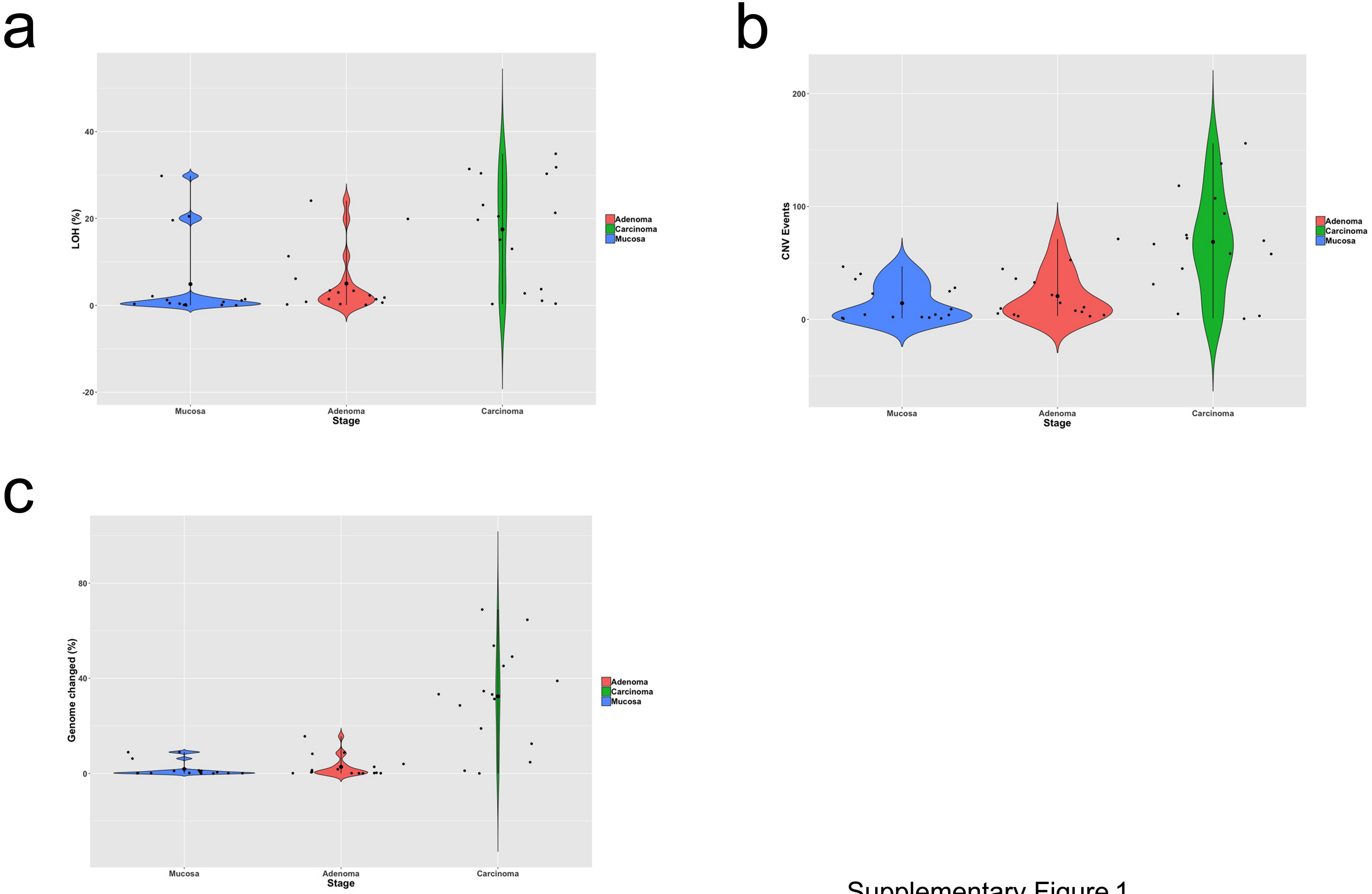

Supplementary Figure 1

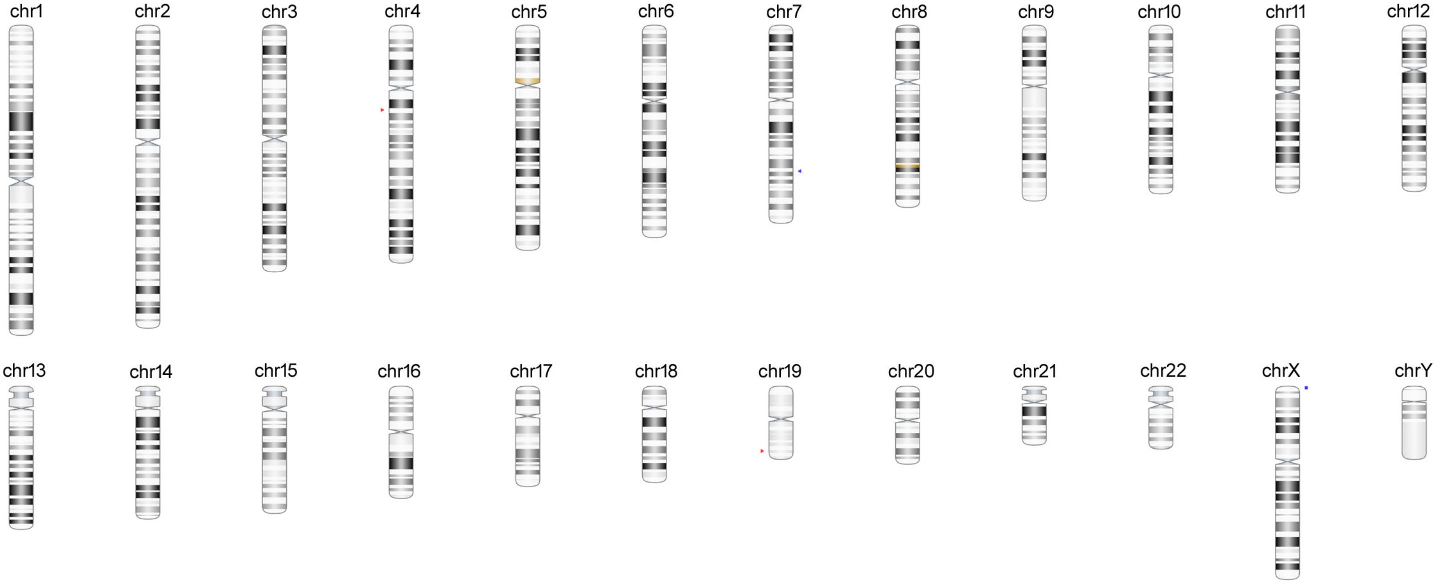

Supplementary Figure 2

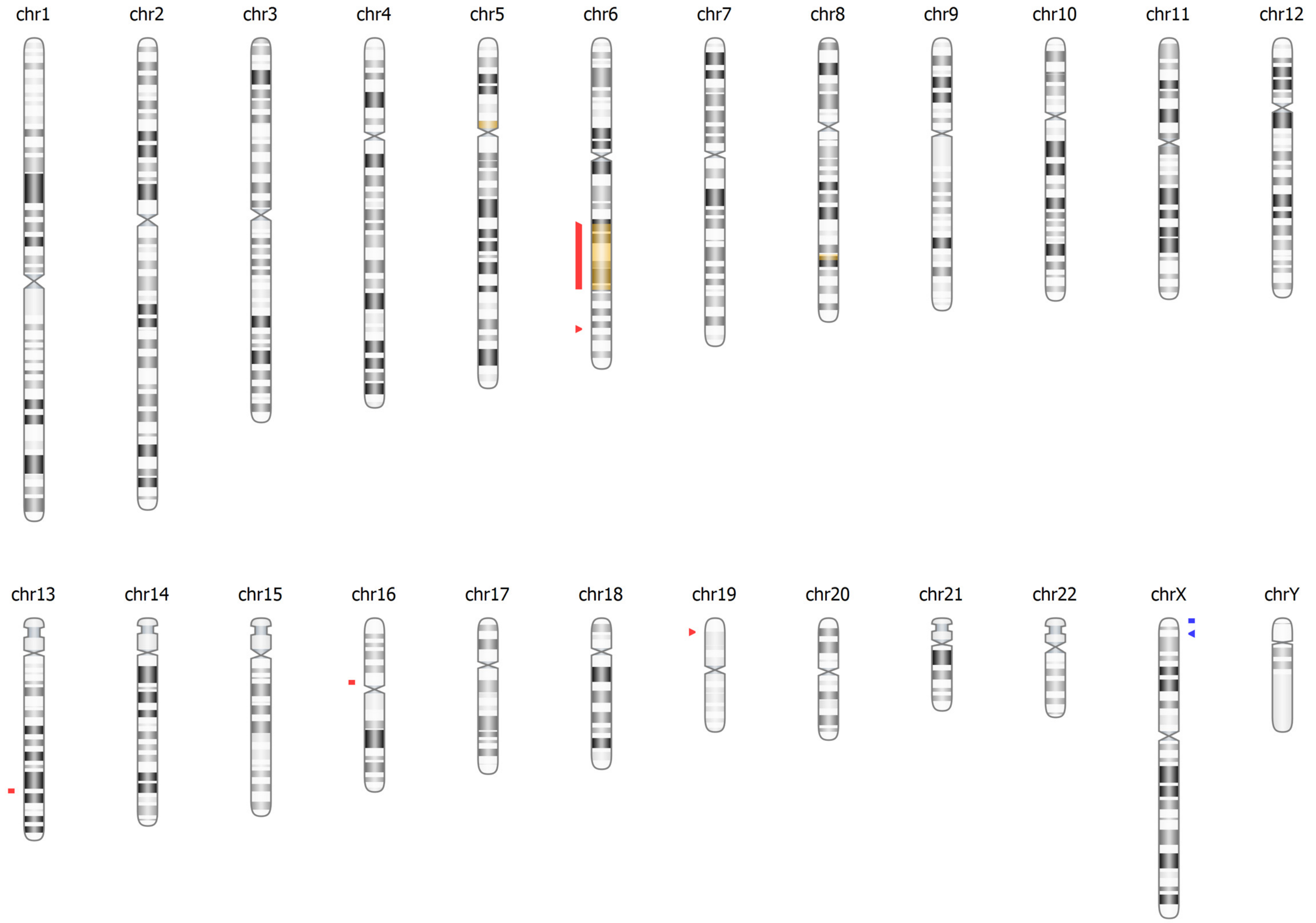

Supplementary Figure 3

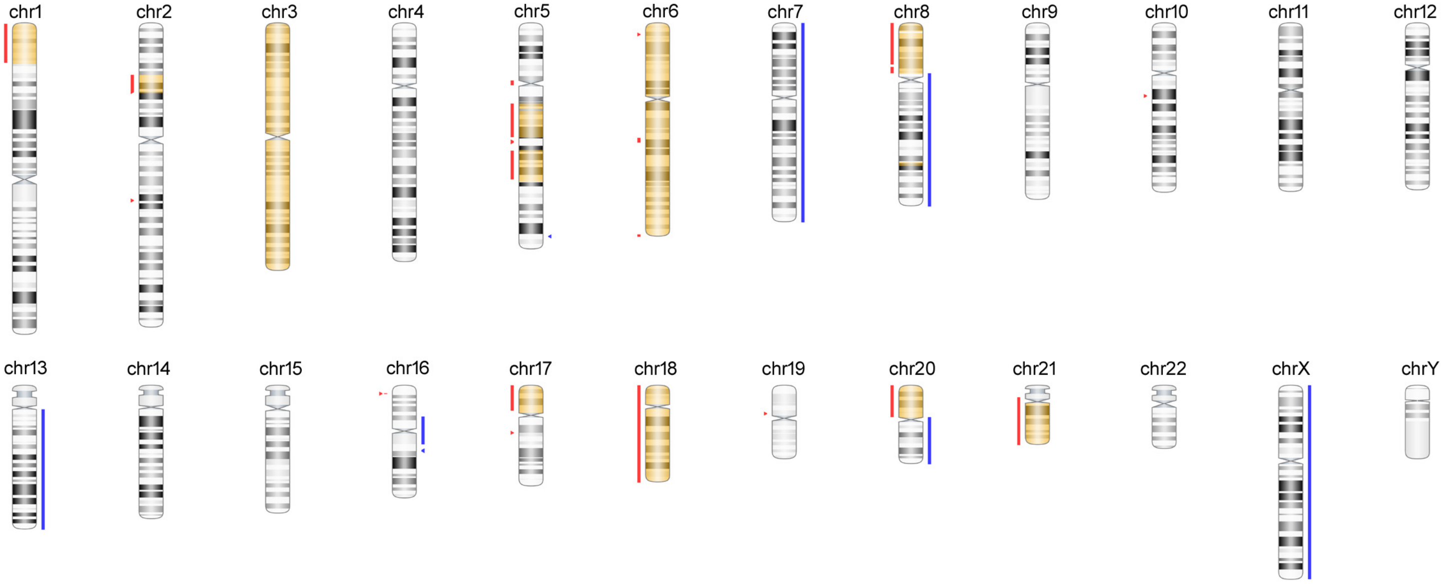

Supplementary Figure 4

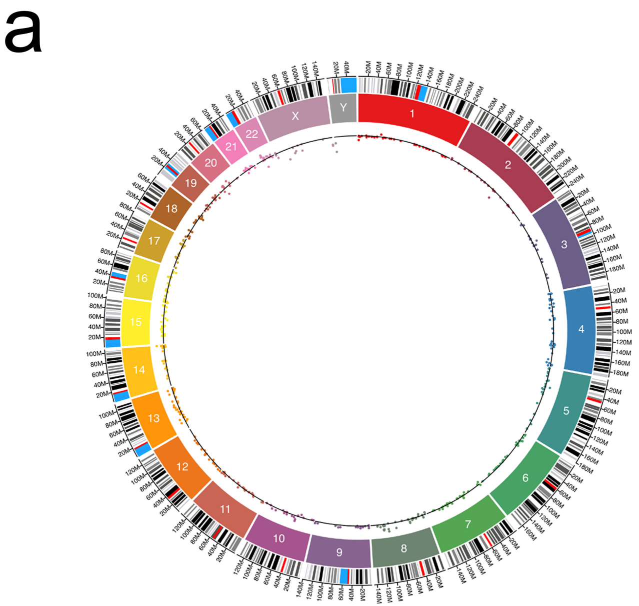

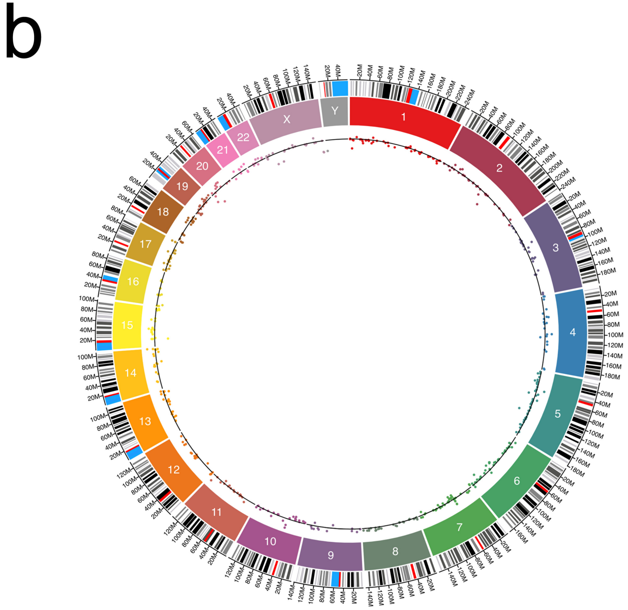

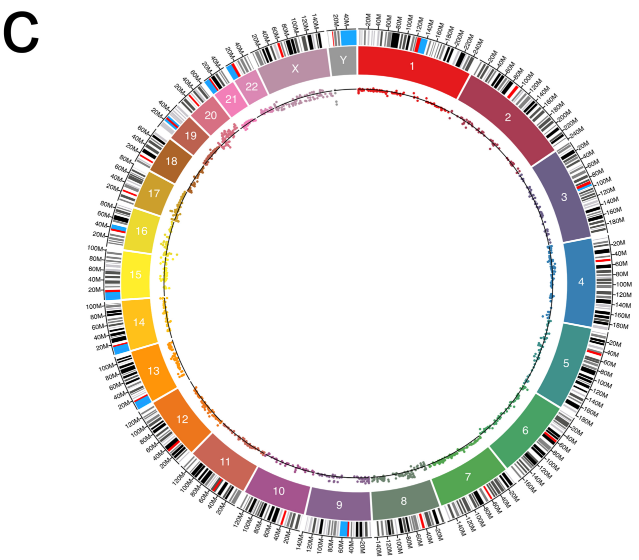

Supplementary Figure 5
