# Supplementary material for: Overexpression of the cohesin-core subunit SMC1A contributes to colorectal cancer development
Source: J Exp Clin Cancer Res. 2019 Mar 1;38:108. doi: 10.1186/s13046-019-1116-0 (PMC6397456; doi:10.1186/s13046-019-1116-0)
Supplement: Supplementary file 6 — Table S6. SMC1A synonymous variants identified in carcinoma samples. (PDF 30 kb) [file 13046_2019_1116_MOESM6_ESM.pdf]

Supplementary Table 6. *SMC1A* synonymous variants identified in carcinoma samples.

Subject	Nucleotide change	Amino acid	Stage
2	c.A1350G	E450	carcinoma
5	c.C165T	N55	carcinoma
5	c.C1631T	A544	carcinoma
9	c.A942G	A314	carcinoma
10	c.C978T	Y326	carcinoma
12	c.A942G	A314	carcinoma
