# Supplementary figures and images for: Overexpression of the cohesin-core subunit SMC1A contributes to colorectal cancer development
Source: J Exp Clin Cancer Res. 2019 Mar 1;38:108. doi: 10.1186/s13046-019-1116-0 (PMC6397456; doi:10.1186/s13046-019-1116-0)

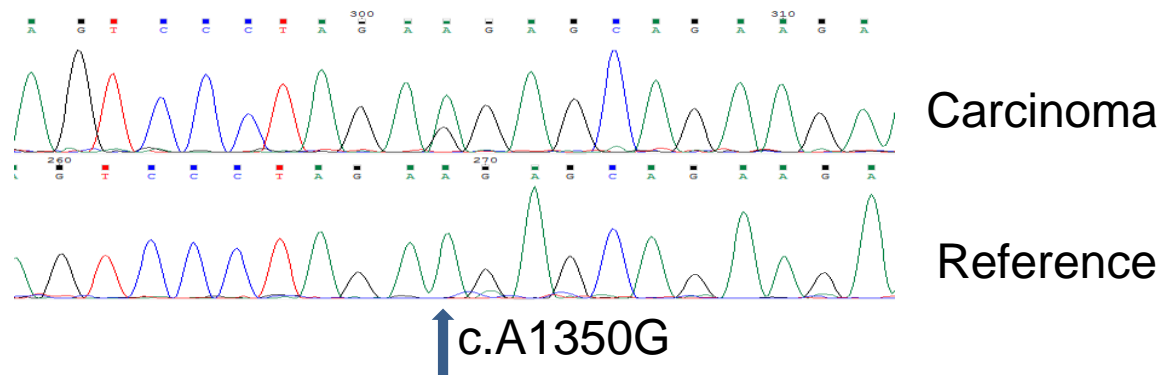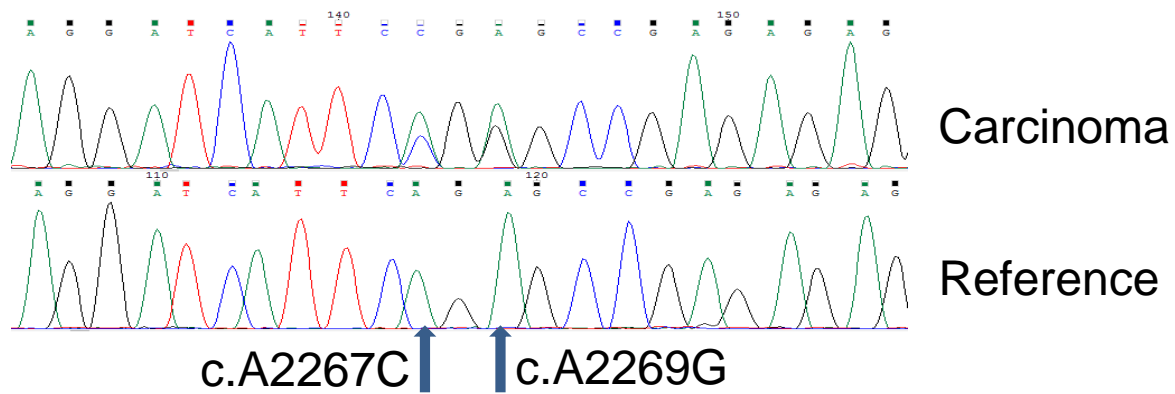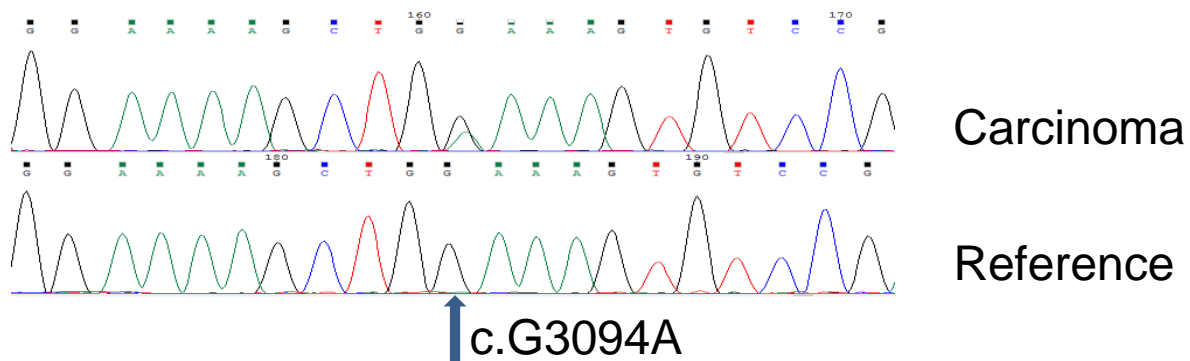

Supplement: Supplementary file 7 — Figure S6. SMC1A mutational screening. Example of representative SMC1A sequencing is reported, showing multiple nucleotide changes in the carcinoma deriving from subject 2. (PDF 64 kb) [file 13046_2019_1116_MOESM7_ESM.pdf]

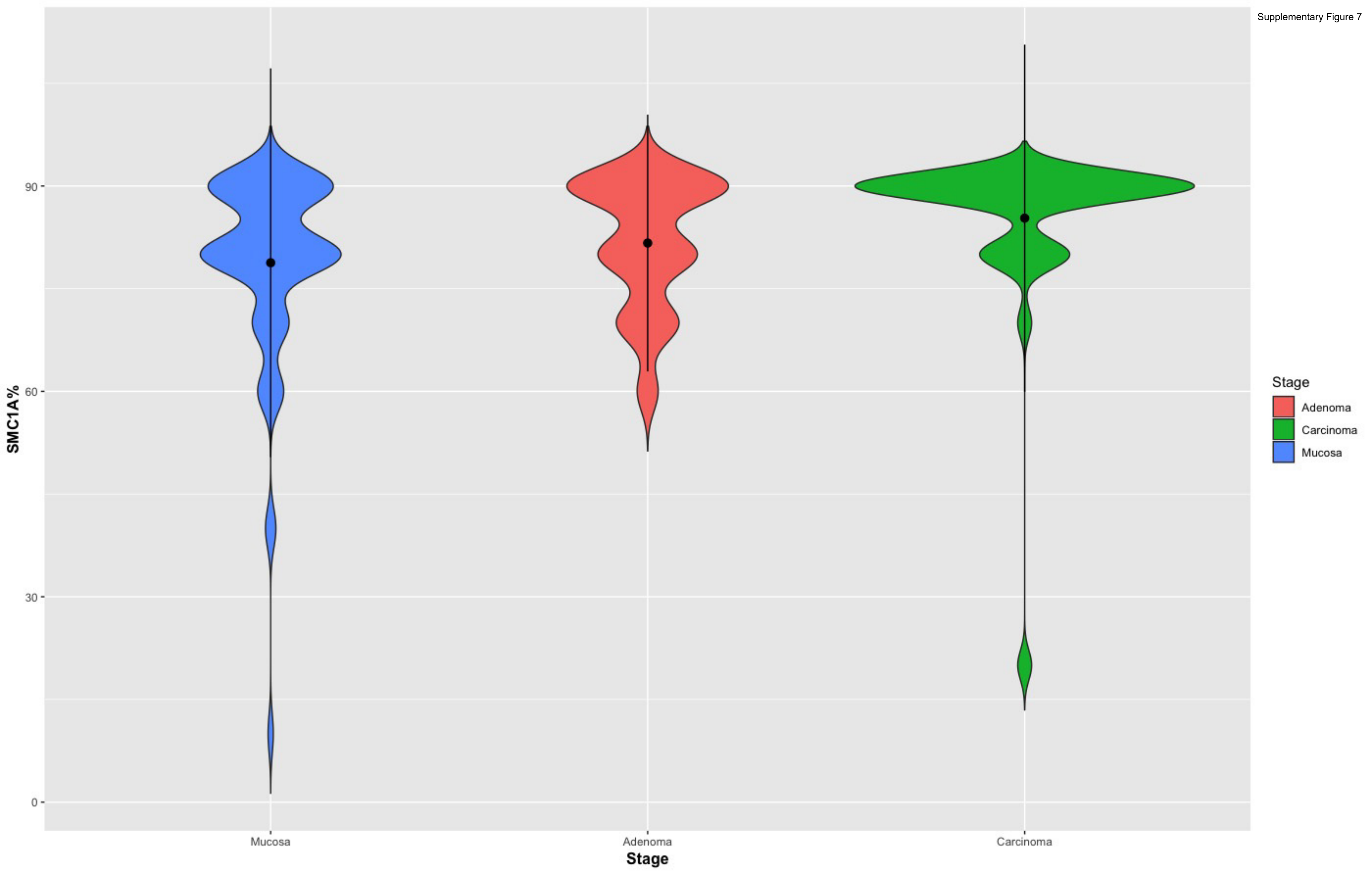

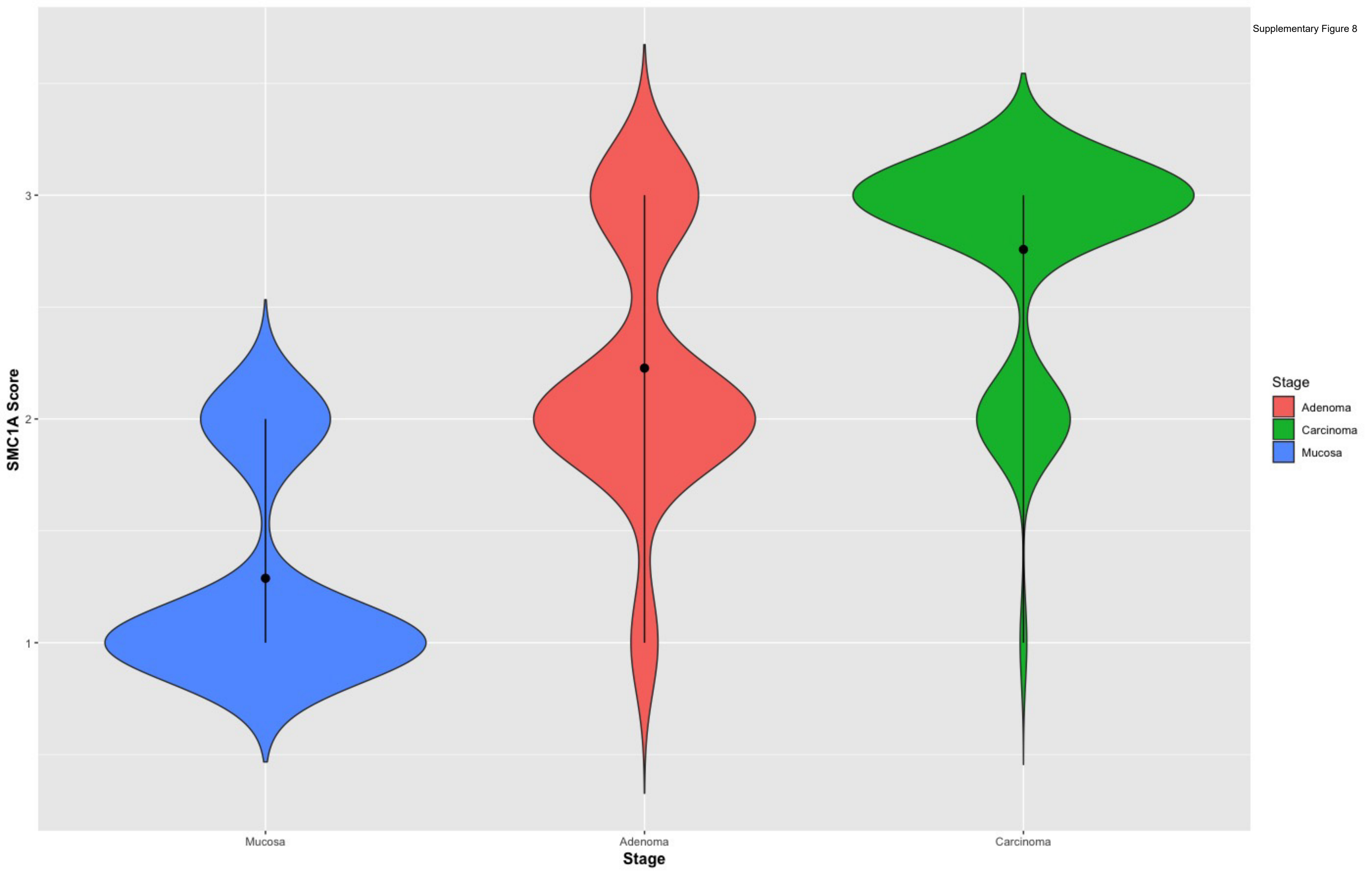

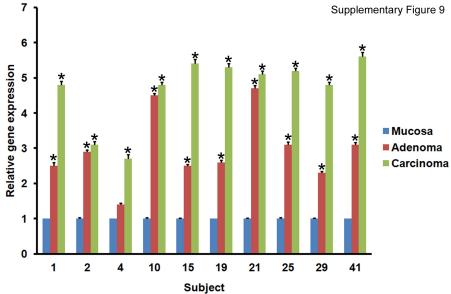

Supplement: Supplementary file 9 — Figure S7. Violin plot showing the distribution of SMC1A-positive cells. Figure S8. Violin plot showing the distribution of SMC1A staining intensity. Figure S9. Immunohistochemistry data was validated by RT-qPCR. *p < 0.05. (PDF 1153 kb) [file 13046_2019_1116_MOESM9_ESM.pdf]

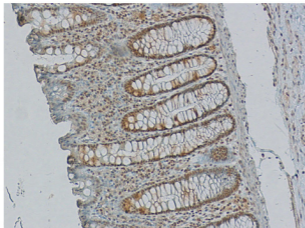

Mucosa

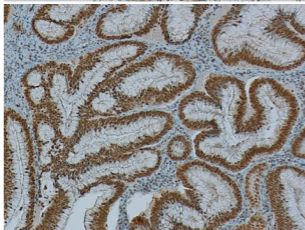

Adenoma

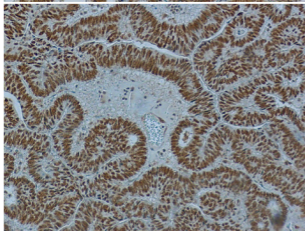

Carcinoma

Supplement: Supplementary file 10 — Figure S10. SMC1A immunohistochemistry in mucosa, adenoma and carcinoma (PDF 1996 kb) [file 13046_2019_1116_MOESM10_ESM.pdf]

a

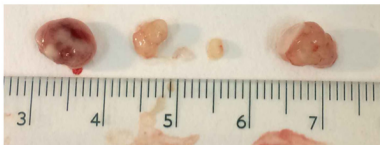

b

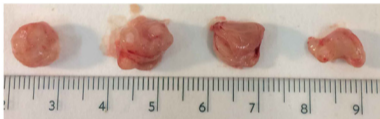

c

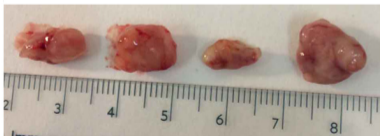

Supplementary Figure 11

Supplement: Supplementary file 11 — Figure S11. Effects of SMC1A mutation and overexpression in in vivo. (a) Representative images of tumours formed in the mice with HCT116 cells. (b) Tumours formed in the mice in which HCT116 SMC1A wild-type cells were implanted. (c) Tumours induced by HCT116 SMC1A c.A2027G cells. (PDF 1199 kb) [file 13046_2019_1116_MOESM11_ESM.pdf]

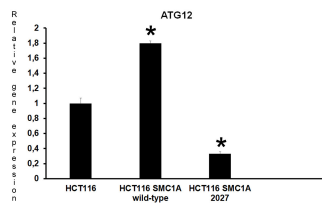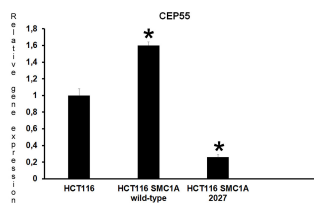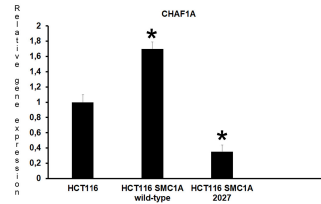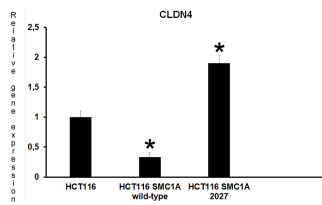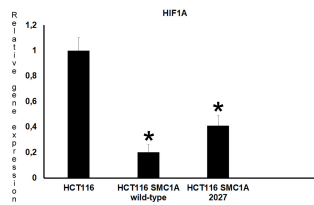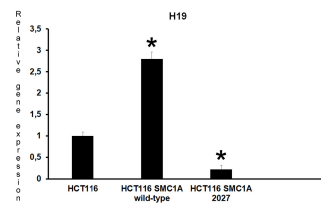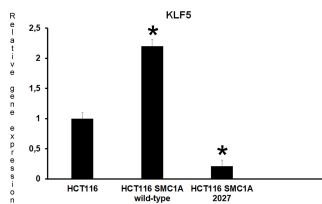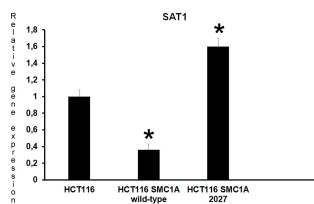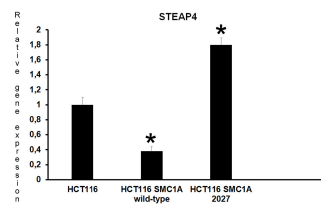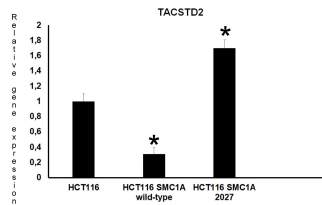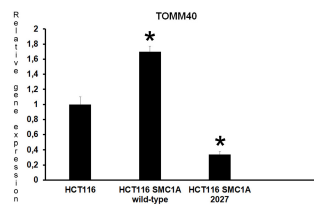

Supplementary Figure 15

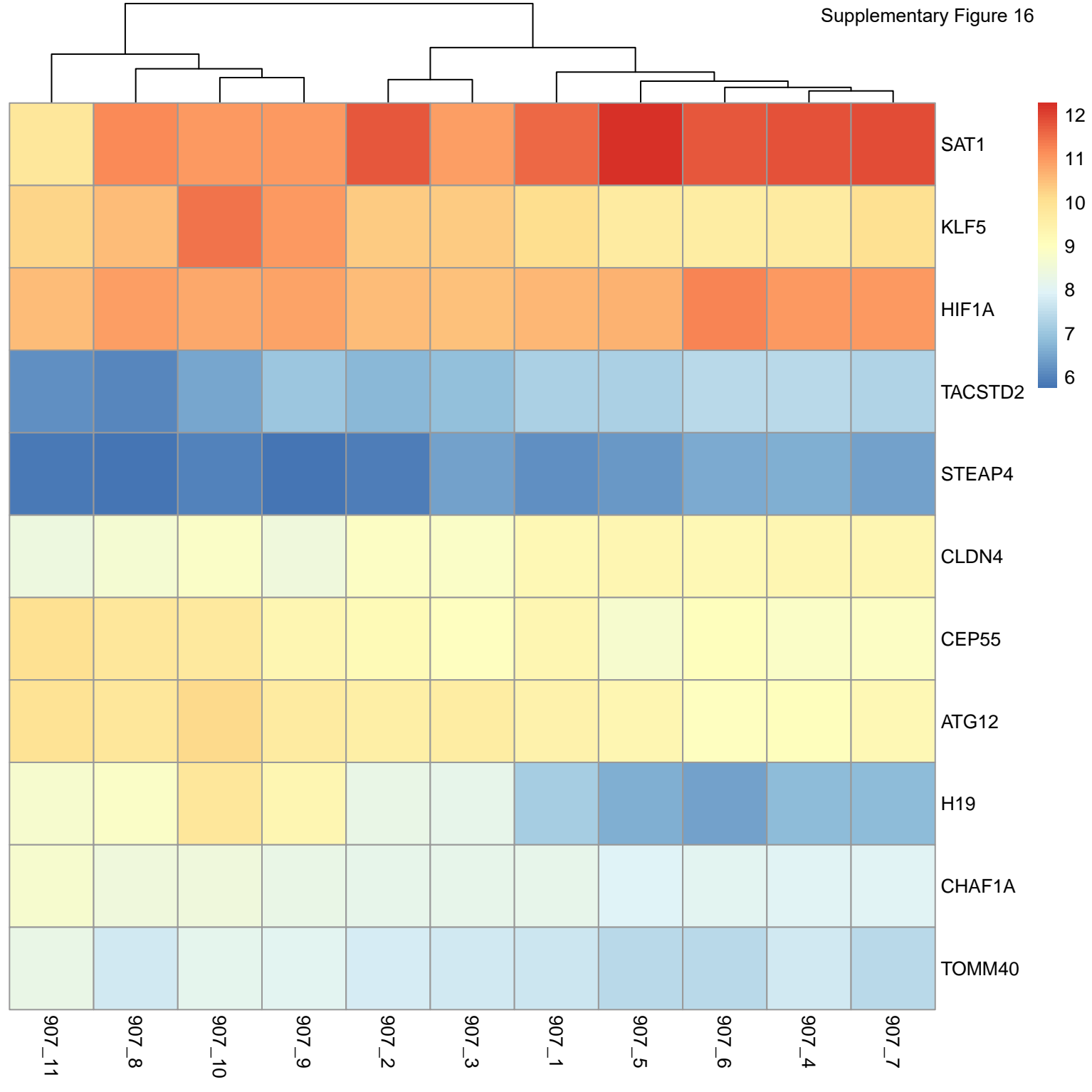

Supplement: Supplementary file 14 — Figure S15. Dysregulated genes in induced tumors. RNA-seq data was validated for eleven genes, ATG12, CEP55, CHAF1A, CLDN4, H19, HIF1A, KLF5, SAT1, STEAP4, TACSTD2 and TOMM40, by RT-qPCR. *p < 0.05. Figure S16. Heatmap of the eleven dysregulated genes validated by RT-qPCR. (PDF 3904 kb) [file 13046_2019_1116_MOESM14_ESM.pdf]
