# Supplementary material for: Overexpression of the cohesin-core subunit SMC1A contributes to colorectal cancer development
Source: J Exp Clin Cancer Res. 2019 Mar 1;38:108. doi: 10.1186/s13046-019-1116-0 (PMC6397456; doi:10.1186/s13046-019-1116-0)
Supplement: Supplementary file 8 — Table S7. Prediction of mutation effects on SMC1A protein using Mutation Tester and PolyPhen2. (PDF 17 kb) [file 13046_2019_1116_MOESM8_ESM.pdf]

Supplementary Table 7. Prediction of mutation effects on SMC1A protein.

| **Amino acid change** | **Effect (Mutation Tester)** | **Effect (PolyPhen2)** |
|---|---|---|
| **Adenoma** | | |
| S14P | Probably deleterious | Probably damaging |
| D207G | Probably harmless | Benign |
| S653P | Probably deleterious | Probably damaging |
| I849V | Probably harmless | Benign |
| N888D | Probably harmless | Benign |
| D1063N | Probably harmless | Benign |
| L1141F | Probably harmless | Probably damaging |
| **Carcinoma** | | |
| I299T | Probably deleterious | Probably damaging |
| K317R | Probably deleterious | Probably damaging |
| V343M | Probably deleterious | Probably damaging |
| Q483R | Probably deleterious | Probably damaging |
| T535I | Probably deleterious | Probably damaging |
| G643E | Probably deleterious | Probably damaging |
| A656T | Probably deleterious | Probably damaging |
| E676G | Probably deleterious | Benign |
| Q756P | Probably deleterious | Probably damaging |
| S757G | Probably harmless | Benign |
| D833G | Probably harmless | Benign |
| M935T | Probably deleterious | Probably damaging |
| M935V | Probably deleterious | Probably damaging |
| E1032K | Probably deleterious | Probably damaging |
| F1182L | Probably harmless | Benign |
