# Supplementary material for: Overexpression of the cohesin-core subunit SMC1A contributes to colorectal cancer development
Source: J Exp Clin Cancer Res. 2019 Mar 1;38:108. doi: 10.1186/s13046-019-1116-0 (PMC6397456; doi:10.1186/s13046-019-1116-0)
Supplement: Supplementary file 12 — Figure S12. Classification of three tumors deriving from the inoculation of HCT116 (907_1, 907_2 and 907_3, red circle), four deriving from HCT116 overexpressing SMC1A wild-type (907_4, 907_5, 907_6 and 907_7, blue circle) and four deriving from HCT116 harboring SMC1A c.A2027G mutation (907_8, 907_9, 907_10 and 907_11, green circle) by gene expression. Table S8. Dysregulated genes in HCT116 SMC1A wild-type induced tumors. Table S9. Dysregulated genes in HCT116 SMC1A c.A2027G induced tumors. (PDF 851 kb) [file 13046_2019_1116_MOESM12_ESM.pdf]

Supplementary Figure 12

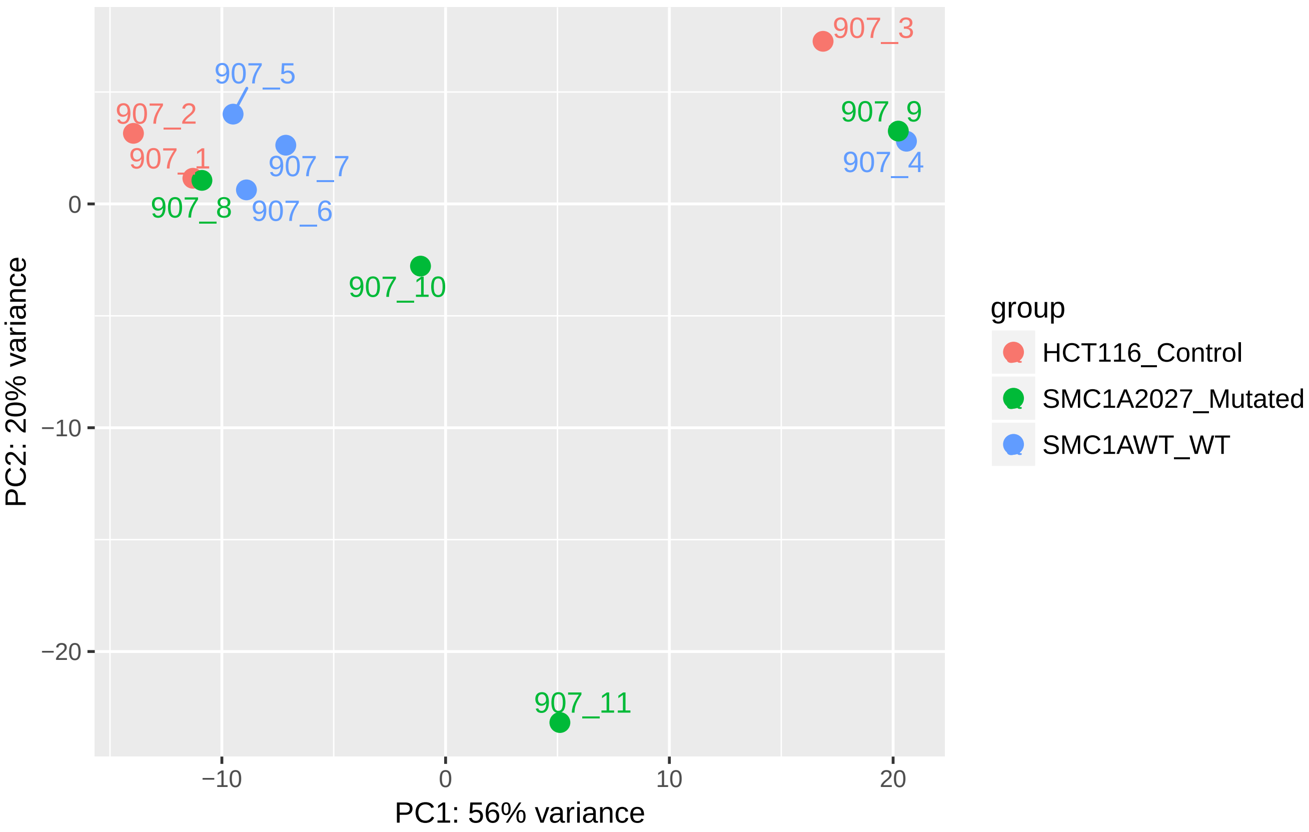

Supplementary Table 8. Dysregulated genes in HCT116 SMC1A wild-type induced tumours.*

GENE	LogFoldChange
AAGAB	0.257195505268628
AAK1	-0.259209914505657
AARS	0.275755961297007
AATK	0.317743470447403
ABCA7	0.328425958812578
ABCC9	-0.296818341831637
ABCE1	0.189115907721298
ABR	-0.232414568605627
ACAT2	0.370296611445516
ACLY	0.342525357570985
ACP5	-0.424234730324524
ACSL1	0.34623272557512
ACTN2	-0.305073134774558
ADAM19	-0.391753344872023
ADRA2C	0.30463519288772
ADSSL1	-0.504075226628719
AEN	0.35100928102763
AFF1	-0.299472854413335
AFMID	0.311909282690368
AGAP2	-0.32199476323605
AHR	-0.300824739838227
AIG1	-0.27600989295451
AIM1	-0.448431553816241
AKAP1	0.38323060391067
ALDH1A2	0.187012770890331
ALG10B	-0.3502405022552
AMOT	-0.455509916947733
ANK3	-0.412806957520431
ANKH	-0.401600484311432
ANKIB1	0.226469831741101
ANKRD12	-0.282681039199523
ANKRD13C	0.317683634527258
ANKRD50	-0.257774971678996

ANKRD6	-0.366587624317033
ANTXR1	-0.397892247662371
AOC1	-0.400216913742196
APBB2	-0.266750603164767
APEX2	0.318130158287071
APOBEC3F	-0.40609520975204
APOBEC3G	-0.351912500353647
APP	-0.279391667650687
ARHGAP15	0.204407220325916
ARL4C	-0.807044507203783
ARMC6	0.332478391863256
ARSD	-0.328180137673103
ASNS	0.266555393006516
ATAD3A	0.36276530394268
ATAD3B	0.508094005063599
ATF7	-0.266602494262384
ATIC	0.213412715205768
ATL1	-0.360225248147336
ATP1B1	-0.319290349323386
ATP5G1	0.425584837253913
ATP5SL	0.267708195497243
ATP6V0D2	-0.350598582220353
ATP7A	-0.272816936127816
AURKB	0.257454209308817
B3GALT2	0.1528112162524
BAIAP3	-0.379826797282775
BAMBI	0.378974461890319
BANK1	-0.514483020939182
BAX	0.332263634554294
BAZ2B	-0.29504286972829
BBS9	-0.365322718674895
BBX	-0.285655153823905
BCAM	-0.381390200796552
BCCIP	0.260969916561289
BCL6	-0.325194469570686
BCL9	-0.239762306870723

BCORL1	-0.378896137789005
BCS1L	0.32477020182322
BMP8B	-0.354176158567242
BMPR2	-0.335160468307665
BVES	-0.731955827389588
C10orf2	0.292034095069476
C12orf60	-0.336235911211453
C16orf45	-0.3204572109994
C17orf96	0.372383487092423
C19orf10	0.374631608489662
C21orf59	0.316900609894715
C3orf55	0.223195164631308
C4orf32	-0.396209947058403
C7orf25	-0.374671198171987
C9orf3	-0.35803833688883
CALB1	0.423033976519607
CALCOCO1	-0.395081168040385
CALCRL	-0.37530497652212
CAMK2D	-0.299406555941959
CAMK2N1	-0.291642729000945
CAPN15	0.490416383803443
CARD11	0.301295426208369
CARS2	0.410271628131444
CASC21	0.154531590497367
CASP4	-0.426316260984571
CCAR2	0.255567047348368
CCDC120	-0.335090578809224
CCDC171	-0.36440326778678
CCDC7	-0.32413204266089
CCDC71L	-0.382258851651031
CCL5	0.190821410698267
CCNE2	0.27798867931557
CCNH	-0.259874814928516
CD109	-0.288469706016845
CD36	0.440399202981817
CD59	-0.267952318441605

CDC123	0.260900073314936
CDC25A	0.279386988419022
CDC42EP3	-0.28385230165305
CDC6	0.311970362551564
CDH15	-0.315176932535032
CDH3	-0.265862096625495
CDH5	-0.27905624569307
CDIP1	0.337041053328545
CDKL5	-0.333938342507348
CDR2L	0.348739482987008
CDX2	0.344542328730084
CEMIP	-0.29387585555453
CENPW	0.322942101151209
CFLAR	-0.35176398786478
CGREF1	-0.312132939873099
CHD3	0.264412208174053
CHD6	-0.407955654766142
CHKA	0.408988895816043
CHPF	-0.400554176006606
CHPT1	0.32974282207869
CHRNB4	-0.346356970758761
CHST2	-0.396627751868336
CLDN3	-0.329393903341546
CLIP4	-0.313085169570064
CLMN	-0.258659458081901
CLPTM1L	0.220135128417881
CLSTN1	-0.208881487034634
CMAHP	0.225560804086764
CMC2	0.364860274961563
CMTM8	-0.314960712386703
CNTN1	-0.418999979132517
COL18A1	-0.342674294509519
COL4A4	-0.420832544778452
COPS3	0.286237620722999
CREB3L2	-0.1714509066321
CSGALNACT1	-0.401147964329454

CTBS	-0.362642807239314
CTGF	-0.401259718129015
CTHRC1	-0.365456988605039
CTIF	-0.326186501242525
CXXC4	-0.357615254293887
CYC1	0.301154505831709
CYP51A1	0.292448741588535
DANCR	0.258564661080187
DAPK1	0.336402136181401
DAZAP1	0.256786497968883
DCAF10	-0.238115986254263
DDN	0.322338752981314
DDX21	0.183142748717864
DDX27	0.390087367768139
DHCR7	0.498567690617035
DHX37	0.39371136888326
DIRAS3	-0.356580876577708
DLG4	-0.337268958480129
DNAJC4	-0.347639975325891
DNER	-0.552161901360006
DOCK9	-0.230163836476576
DOHH	0.319334334642219
DPEP1	0.387970292602922
DPH2	0.396080693967835
DPH5	0.267420563983902
DPP4	0.461949675725578
DSC2	-0.345790943397984
DSC3	-0.953520584150088
DSG2	-0.237225791445901
DST	-0.274597987810162
DTNB	-0.31553061230249
DUSP6	-0.642105998712446
DUSP9	0.349291514970916
DVL2	0.339653827987389
E2F8	0.326381549186617
EBNA1BP2	0.321953173081668

EDAR	-0.242395832166296
EDIL3	-0.319030631416905
EFNB1	0.340985222842039
EGFR	-0.350345450028773
EI24	0.2608592399309
EIF2B4	0.291341752533544
EIF3L	0.218768994129938
EIF4G1	0.181960131947056
ENDOD1	-0.330823728878296
ENPP1	-0.293772214972203
EPB41	-0.306783704567434
EPB41L1	0.229228315905988
EPB41L4A	-0.52481672292793
EPGN	-0.271664183243779
EPHA4	-0.409302422169598
EPS8	-0.420873936962443
ERAP1	-0.37722867502214
ERV3-1	-0.445322916063965
ESPN	-0.298778746802322
ETS2	-0.310233021840904
ETV5	-0.313108717768018
ETV6	-0.44183071570444
EVPL	-0.280288288917123
EXO1	0.309186282037941
EXOSC2	0.325604211406817
EXOSC7	0.30774318947293
FADS1	0.240618239226551
FAM102B	-0.266662527930424
FAM107B	-0.268081763262497
FAM129A	-0.425370287157921
FAM136A	0.325399672525307
FAM149B1	-0.315192093921688
FAM160A1	-0.350247653610879
FAM168A	-0.334227430479965
FAM168B	0.263192491045555
FAM73A	-0.291818645448864

FAM98A	0.252099119754972
FARSA	0.286458882647516
FAT4	-0.359372749017622
FBL	0.347745384809143
FBXO25	-0.306638660048313
FDFT1	0.533855066394451
FDPS	0.442535197316768
FGFBP1	0.560711084241693
FHOD3	-0.399759972312362
FKBP10	-0.353888540765525
FNDC3A	-0.210307475974541
FOXC1	-0.360717291919431
FOXJ2	-0.229895660528208
FUT8	-0.420361548555783
GAL	0.311822763081095
GALNT18	0.302011738839186
GART	0.450262014607657
GATA6	-0.384138559335309
GCN1L1	0.263036131602262
GCNT3	0.319919725579332
GEMIN4	0.246459176229852
GEMIN5	0.409856077074342
GLG1	-0.246481879588226
GLI2	-0.422995281814268
GLOD4	0.281981759041368
GLT8D2	-0.432970959235962
GNA13	-0.183194442666184
GNL3	0.360403025838563
GOT1	0.312052179525158
GOT2	0.287200064075466
GPC4	-0.502365412562771
GPR155	-0.41486674595937
GPR176	-0.28963601788076
GPRIN1	0.279204129842352
GPRIN3	-0.485676758927356
GRAMD1C	-0.360291895379658

GTPBP4	0.213164507889711
GUF1	0.314088499497573
HBE1	-0.34221235147982
HDAC9	-0.571961017131127
HDGF	0.197514809070704
HEATR1	0.298624713133005
HEATR2	0.236812790101887
HIAT1	0.26992681055591
HINT3	-0.284520855393745
HIPK2	-0.245085703823149
HIST1H2AB	0.323111782898048
HIST1H4B	0.324315047060605
HIST1H4H	0.332311919315903
HIVEP3	-0.461401920646205
HMGCR	0.310099813039265
HMGCS1	0.662282566320652
HNF4A	0.610763528541728
HNRNPA1L2	0.336393845898903
HNRNPH3	0.301165948406028
HSD17B1	0.312872860393637
HSPH1	0.323224634480135
HTR7	0.389867067728204
HTRA2	0.344309419232055
HYAL3	0.346425768917966
IARS	0.229059088217272
IDI1	0.376064334093267
IDS	-0.322492490343524
IFRD2	0.324366521579996
IGF1R	-0.434136415571556
IGHMBP2	0.41843879309082
IKZF2	-0.397058347393019
IL17RD	-0.340417966670502
IL31RA	-0.224183891149059
INHBB	-0.31738108859941
INPP4B	-0.498333939861476
INSIG1	0.57225242618358

INSR	-0.238763920992794
ITGA2	-0.267754666582377
ITGA3	-0.216246103274383
ITGB8	-0.616490870370596
ITM2B	-0.429589993008572
ITM2C	-0.237301287480349
ITPK1	0.243443352830973
ITPR2	-0.273319158778295
ITPR3	-0.259926120107556
JOSD1	0.232343809578644
KARS	0.256635071902237
KAT2A	0.30936789257325
KAT6B	-0.287941783073261
KATNAL1	-0.274034207949398
KCNH3	0.238882357700156
KCNJ12	0.314044448509362
KCNJ2	-0.316338634817345
KCNK9	-0.342761128627272
KDSR	-0.265568915848245
KIAA0100	0.258522719126662
KIAA0355	-0.278332243735125
KIAA0430	-0.215386392267727
KIAA1109	-0.319349269122758
KIAA1161	-0.307730745466755
KIAA1462	-0.498634463812353
KIF13B	-0.304907452954726
KIF5C	-0.325325966868366
KLF12	-0.490244513121331
KLHL5	-0.374553593891
KLK6	-0.40215086968514
KLK7	-0.444217757490484
KRT14	0.332045396330618
KRT17	0.412661700970999
KRT23	-0.342912681511318
KRT4	0.177857822891364
L1CAM	-0.387477476531504

LAMB1	-0.307372799665975
LAMP2	-0.258503148674657
LBX2	-0.317408965522914
LCAT	0.219812551535849
LCOR	-0.317484361769666
LCORL	-0.358459616581006
LEPR	-0.357421128677142
LETM1	0.308107009633409
LHX5	0.267758975109371
LIMCH1	-0.552554607646358
LINC00592	-0.302782011295304
LINC00669	-0.309486030784143
LMBRD1	-0.277487489706672
LOC730102	-0.384950349234989
LPAR6	-0.214304456456457
LPCAT2	-0.328958457184824
LPIN1	0.306182308720482
LPL	0.39416368528743
LPP	-0.277217910596227
LRRC59	0.209189540602028
LSM7	0.378266031401137
LTBP3	-0.456165691634522
LTV1	0.286337384603532
LY6D	0.361399248994698
LYN	0.319485227043106
MACC1	-0.417753505278593
MAML2	-0.656717727044327
MAN1A1	-0.237770225073471
MAN2B2	-0.355220341255484
MAP2	-0.346004625003361
MAP3K1	-0.295583956427299
MAP3K13	-0.265292675878596
MAP3K5	-0.258087687588202
MAST4	-0.366393570570354
MAT1A	-0.390621763883667
MATN2	-0.376799972765778

MBNL3	-0.735831655177537
MCM10	0.236793695407531
MCOLN3	-0.368588630150577
MDN1	0.267238940325519
MEA1	0.305156404221312
MEF2A	-0.264980874820387
MEF2C	-0.413096799324332
MEIS2	-0.327019731357942
MFN2	0.18756614780069
MGAT1	0.253646263114823
MGAT4A	-0.392689782602891
MISP	0.440311809640813
MLXIP	0.262968521378506
MMP13	-0.511976068319114
MMS19	0.295244053583926
MPDU1	0.278988500148708
MRC2	-0.35642494214737
MRM1	0.31323545131557
MRPL2	0.353744378644279
MRPL47	0.288686925377242
MRPS2	0.270006730247582
MRPS27	0.20718029237228
MRTO4	0.341773030326097
MSLN	-0.292527606570497
MT1E	-0.41255909667121
MT2A	-0.328935733302526
MTA3	-0.231394853759568
MTHFD2	0.291725122074417
MTMR10	-0.282080682910882
MUM1L1	-0.467701584418817
MVD	0.59686433662287
MVK	0.387979586823337
MYBL2	0.320842240307653
MYLK	-0.347446097110236
MYO1D	-0.287272161200785
MYO6	-0.253911662542727

NADK	0.293148940011098
NARS	0.331874802181501
NCLN	0.260623141286942
NCMAP	-0.347171593470575
NCOA3	-0.232428531526654
NCOA7	-0.34475742530724
NDUFAF2	0.41484609142773
NDUFB9	0.271487950294719
NDUFS3	0.249044059066993
NEDD4L	-0.265765641736247
NELFA	0.259781850728877
NEO1	-0.316736135663626
NES	-0.339984889151504
NET1	-0.292100048595723
NFAT5	-0.37326364385186
NFATC2	-0.302537642424023
NFIA	0.371252630735864
NFS1	0.361102845269564
NHS	-0.48411483203896
NHSL1	-0.358267153845178
NIPSNAP3A	-0.323685509550688
NLE1	0.368947446145257
NOL4L	-0.428507478994152
NOP16	0.39855818206197
NOP56	0.319187001278092
NOS1	-0.186871067358832
NOTCH3	-0.288230840221759
NPM3	0.417911168042869
NPTXR	-0.32132219633896
NR2C2	-0.255284050741543
NSMF	0.251403214622915
NT5E	-0.329446250792803
NTHL1	0.367520124281651
NUMA1	-0.268631483905481
NUP85	0.26950865626432
NUP88	0.260427571569145

NUP93	0.271985482616293
ODC1	0.29666829300021
OR51B5	-0.357778956270472
ORC1	0.266578227253527
ORC6	0.370821341897704
OS9	-0.294705853510904
OSBPL10	-0.3755448947767
OTUB2	-0.332452710795969
PACSIN1	-0.340332347947638
PAK1IP1	0.303972036285
PALLD	0.367403753610032
PAQR8	-0.415347920391825
PARK7	0.238955466489735
PARP4	-0.18268668736799
PARP8	-0.278121165900193
PARVA	-0.256070977935809
PASK	0.437350850522596
PBRM1	-0.243959866780905
PCDH1	-0.372924207520404
PCDHGA10	-0.3344992979216
PCDHGB2	-0.476246807494925
PCLO	-0.27809876523534
PCNA	0.302208033491773
PCSK9	0.323988570410763
PDCD11	0.22626876419687
PDCD2	0.26278817170419
PDCD2L	0.359548561879953
PDHA1	0.212351240003052
PDRG1	0.382966486762729
PDXK	0.230628039196501
PEAK1	-0.305199602549576
PEG10	-0.379651780048928
PFAS	0.483581338771645
PGAM5	0.433630303449444
PGM2L1	-0.401433499124968
PHB2	0.276209023672395

PHF5A	0.328091619307062
PHLDA1	-0.242355951114431
PHLPP1	-0.289818307517755
PI15	0.293573495253884
PIK3CA	-0.233242519618866
PIK3R1	-0.417649041833526
PLBD1	0.282728615785825
PLCB1	-0.313880027397478
PLD1	-0.374342409363191
PLEKHA3	-0.420544522624506
PLEKHA5	-0.385709348381763
PLEKHB1	-0.475373276478429
PLEKHS1	-0.226964193075027
PLSCR4	-0.459954201614126
PML	-0.263445642227687
PMPCA	0.263987867086554
PNMA2	-0.533758841824297
PNP	0.386143863246609
POLD2	0.225401839618434
POLG	0.426554214033186
POLI	-0.31491497179535
POLK	-0.280223873031834
POLQ	0.386151399887776
POP7	0.294402829862165
POPDC3	-0.451993383451342
PPFIA3	0.360667357848455
PPFIBP1	-0.26918965336027
PPID	0.279249104061936
PPL	-0.317077469613974
PPM1A	-0.217184076773184
PPP4C	0.26819191778341
PPRC1	0.291014367543528
PREP	-0.240312736519653
PRKAA2	-0.574751871754205
PROCR	0.325459285298083
PRPF19	0.179281349547704

PRR5	0.327981751380068
PRSS16	-0.316772938988223
PRSS35	0.267765211640351
PSAT1	0.303901760921688
PSMB6	0.281232948646175
PSMC5	0.232187055793464
PSMG1	0.337660711273998
PTDSS1	0.263607453441468
PTP4A1	0.201174585514735
PTPN12	-0.273681946702202
PTPN14	-0.403956986663434
PUS7	0.260780185789483
PWP2	0.336146733876765
QARS	0.223885996749286
QRSL1	0.334621454854203
RAB27B	-0.417309739825663
RAB3B	-0.335601597757381
RAC3	0.268712817636024
RAE1	0.26247830620703
RAMP1	-0.391513359807817
RASSF8	-0.553933979513699
RBM14	0.44536991985371
RBM47	-0.33398289342423
RBMS2	-0.283613107021913
RC3H2	-0.218032595562031
RCC1	0.287153252664589
REEP3	-0.349635805443165
RELN	-0.302202532713878
RICTOR	-0.224013238297109
RND2	-0.361794300708613
RNF13	-0.304611972296038
RNF167	0.283206028449559
RNF182	-0.657491258126472
RNF213	-0.308332263304271
RNPEP	0.209746471168291
RPARP-AS1	0.325601304277711

RPL13A	0.386063774809793
RPL27A	0.248826115768166
RPL9	0.360539921915688
RPP30	0.237512462206552
RPS19BP1	0.424828172195585
RPS6KA2	-0.383821269738466
RPS6KA5	-0.314875746423047
RRAGD	-0.261997356324536
RRP1	0.329450249700764
RRP12	0.314844280283981
RRP1B	0.25511579954798
RRP9	0.401130031445279
RUNX1	-0.580282778084246
RUVBL1	0.288044800071737
SAMD9L	-0.331724533937747
SASH3	0.249030434269955
SATB1	-0.350063384526789
SCD	0.355190776559467
SCRIB	0.467860916406638
SDC1	0.377113389735982
SDCCAG3	0.35725258624545
SDHA	0.257571578706592
SECISBP2L	-0.288684127348336
SEL1L3	-0.253922750112148
SELL	-0.331219332211215
SERINC1	-0.243848342656149
SERPINA1	-0.5559593884637
SERPINA5	-0.476778867446564
SERPINE1	-0.363552735119493
SF3A3	0.300925546493629
SFN	0.334581251797111
SGCE	-0.380656854934592
SGPL1	-0.221417559405489
SH2D2A	0.288126668682774
SH3D19	-0.321801941697024
SHMT2	0.380422084272497

SIX4	-0.290215347365848
SKIL	-0.347937052880552
SLC15A1	-0.419678066743427
SLC19A1	0.298587217396187
SLC24A1	-0.353583760714324
SLC25A1	0.267037320355928
SLC25A19	0.308489435224784
SLC30A4	-0.324085854854446
SLC3A2	0.346074409133857
SLC43A3	0.338886774738131
SLC4A3	-0.343575882921843
SLC6A20	-0.521052528916274
SLC7A11	0.321367825924676
SLC7A5	0.340127226013937
SLC7A6	0.304158832477069
SLIT2	-0.337814820062281
SMURF2	-0.424561637563587
SNAPC3	-0.278183159356428
SNHG17	0.328366467683876
SNHG9	0.339574923138002
SNORA64	0.481570242943669
SNORD17	0.271960771115842
SNORD79	0.359281438630796
SNTB1	-0.372973199137791
SNTG2	0.21092051256907
SNX10	-0.252032608666847
SNX17	0.219515876497741
SNX7	-0.321843518826816
SORBS1	-0.353462415705385
SORL1	-0.396919215412829
SOX9	-0.499879831592265
SPARCL1	-0.301825207741492
SPATS2L	-0.313056488875132
SPCS1	0.244991372393141
SPOP	0.328768597288217
SPR	0.306918350475942

SPRY2	-0.360121114412707
SPTY2D1	-0.263800415608412
SQLE	0.334577511535661
SQRDL	-0.33393367022109
SREBF2	0.238638699209089
SRGAP2D	-0.329977816046465
SSB	0.28141685480925
SSBP3	-0.319646640434142
SSBP4	0.30239357273681
ST7	0.326234958648098
STAM2	-0.220777384419931
STAT2	-0.325614303467127
STAU2	-0.259975534000642
STEAP1	-0.341424468747398
STEAP2	-0.41140798917408
STOML2	0.256925232546333
STOX1	-0.395266039108159
STRA6	-0.293172780457539
STX7	-0.240633721797379
SUCO	-0.267699239531446
SUSD1	-0.314271613837494
SUSD2	-0.389782885128046
SYK	0.282254767478712
SYNE1	-0.478854481759793
SYNE2	-0.495646284875021
TACC3	0.246037145063016
TAF13	0.346261445106549
TALDO1	0.229690905016136
TANC2	-0.249517068409132
TBC1D8	-0.269162942565729
TCEB3	0.230189311691411
TCF7L2	-0.282479201814942
TCP11L2	-0.319619899138034
TELO2	0.372911504042109
TES	0.180446950416861
TFAP2A	-0.445305761368306

TFPI	-0.305859555677713
TFRC	0.469131274264773
TGFB3	-0.371417204590629
THOP1	0.277517879720849
TIAM1	-0.2744903197865
TM4SF1	-0.574928054443921
TM4SF18	-0.353509985259659
TMC6	-0.271726465841084
TMCC1	0.298034300894719
TMEM2	-0.335352193356816
TMEM200A	-0.387202783361154
TMEM263	-0.27656970570446
TMEM38A	0.32967335116552
TMEM55A	-0.361833298626093
TMOD2	-0.337614799037351
TMTC2	-0.306345689051845
TNIK	-0.523880709426942
TNS3	-0.354245929393023
TOMM5	0.260492034554395
TOMM6	0.363552173863407
TONSL	0.391397535483555
TOP3A	0.256471195324791
TOR1AIP2	-0.196193116962297
TOR4A	0.307991813863366
TOX2	-0.322385370606648
TPBG	0.338713218345054
TRAM2	-0.225477868763186
TRHDE	-0.419301371482818
TRIM16	0.59865727771033
TRIM28	0.186689915812786
TRIM36	-0.268455881728742
TRIQK	-0.452146147352487
TRMT1	0.423895847922005
TRMT112	0.372412801929455
TSC22D1	-0.296108915273091
TSPAN1	-0.36864126274666

TSPO	0.302198435513778
TTC19	-0.246044264484168
TTC27	0.28002847071331
TTN	0.361389984679462
TUBA1C	0.261548093297966
TUFM	0.209639976490586
TUSC2	0.388808908729019
TXNL4A	0.33394062355965
TXNRD1	0.329898236905909
TYMS	0.312965488440574
U2AF2	0.258313403944875
UBE2H	0.262769025563709
UBE2J2	0.328913599932
UBE3D	0.452250096988549
UCK2	0.320611682750777
UFD1L	0.264825912271094
UFSP1	0.265884534495606
UNC13A	0.469528515176014
UTP20	0.207878833783141
UTRN	-0.437928555962431
VDAC3	0.256782565371995
VEZF1	-0.249551061885629
VPS13C	-0.334717973466898
VPS41	-0.23372315575253
WASL	-0.280141887091514
WDR3	0.253591543440992
WDR5	0.31659693666664
WDR72	-0.366616400081529
WDR74	0.246748330057817
WDTC1	-0.381121119623827
WIPI1	-0.31293994532032
WNK2	0.426666454804625
WSB2	0.249411093352256
WWC1	0.370552983805355
WWOX	-0.322933054781479
WWTR1	-0.412475785735384

XPO5	0.227635082906978
XPO6	0.177039938163668
XPOT	0.219775502941338
XRN1	-0.294979498960137
YAP1	-0.258521120206483
YARS	0.347096007659935
YLPM1	0.251234971614823
YRDC	0.313683090056556
ZBTB20	-0.27328614353465
ZBTB38	-0.334719563549286
ZBTB41	-0.213757320439169
ZC2HC1A	-0.30688363891717
ZC3H6	-0.255623171629546
ZDHHC20	-0.24300485442678
ZFHX4	-0.239286279349265
ZFP36L2	0.323402958974224
ZFR	0.197642089930925
ZNF169	0.327044685602788
ZNF33A	-0.278230750150349
ZNF33B	-0.340997349478186
ZNF367	0.352955493467617
ZNF615	0.365330498984959
ZNF648	-0.381665818376451
ZNF771	0.353360239862355
ZNF789	0.320180879649692
ZNF792	-0.374877853711027
ZNF860	-0.398538496354151

Supplementary Table 9. Dysregulated genes in HCT116 SMC1A c.A2027G induced tumours.*

Gene	LogFoldChange
ABCA4	0.400592101215397
ABCF2	-0.27841955309202
ABHD17A	0.466321612439955
ACADVL	0.438436754913556
ACBD4	0.397086560735457
ACSF2	0.480881045257957
ADCY6	0.554730732657733
ADCY7	0.34714222458328
ADPRHL2	0.427368188953336
AES	-0.460134389036358
AHCTF1	-0.310003262382099
AHI1	-0.438588380277164
AIM1L	0.608499945581246
AK6	-0.462744751310323
AKAP9	-0.336653623066578
AKT1S1	-0.381601323353291
ALCAM	-0.435367199922471
ALOX5	0.465532951773224
AMZ2	-0.275676907589951
ANKFY1	0.290557180398765
ANKRD17	-0.248268179328874
ANKRD22	0.40686122022985
ANKRD9	0.437321209966669
ANKS3	0.400865756359018
ANLN	-0.420698712876537
ANO1	-0.660576984827703
ANO9	0.436595772010045
ANP32B	-0.384822903802176
AP3B1	-0.348181149664281
AP5Z1	0.411969388700963
APH1A	0.30517584640646
APOL6	0.520401029488569
ARFGAP1	0.368756764257786

ARHGAP11A	-0.337818335352339
ARHGAP29	0.384852240690438
ARHGDIA	-0.495276897075561
ARHGEF10L	0.526213468559583
ARHGEF17	0.416614288623763
ARHGEF18	0.324309508714368
ARHGEF37	0.530652232350986
ARL14EPL	-0.762084288988482
ARL2	-0.589681533076057
ARL4A	0.745010107494648
ARRDC4	0.444915556028485
ASAP1-IT1	0.368015734084773
ASB1	0.520015605959372
ASPM	-0.453079756795645
ASXL2	-0.410169555625694
ATAD2	-0.401689376016788
ATF5	-0.535733403509956
ATG3	-0.377447565067769
ATG4B	0.383046353318648
ATP10B	0.300940729104923
ATP5J2	-0.735954567652196
ATP6V0E2	-0.456792967752984
ATRN	0.566971588987728
ATRX	-0.271325710686215
ATXN2L	-0.472396345742283
AURKA	-0.542518849509988
B4GALNT4	0.435976879691729
BAG4	-0.287750354605995
BAI2	0.684983663754836
BAZ1B	-0.428096430604387
BCL3	-0.446942797798269
BCLAF1	-0.275887250356998
BLCAP	0.411622319618209
BLVRB	0.387880780443725
BMP7	-0.399460686637606
BNIP2	-0.280961697713949

BOD1L1	-0.321828721723967
BRCA2	-0.346181739651106
BRD4	-0.453379063448434
BRIX1	-0.334811727325722
BTF3	-0.464121759871648
BUB1B	-0.277505663709353
C11orf1	-0.455655407977388
C11orf83	0.588562555033604
C12orf76	0.450575969906971
C17orf97	-0.430810934477197
C18orf21	-0.353850802269503
C19orf43	-0.440525409405687
C1orf210	0.429113299051649
C22orf23	0.304604470400147
C7orf63	-0.452701140824932
C9orf69	0.420233230052502
C9orf89	0.535330409553077
CA12	0.455823466232615
CACNA1B	0.431077793590575
CALD1	-0.266898138421109
CALM2	-0.422352667097245
CANT1	0.287530357861594
CASC5	-0.43485470867642
CATSPER2	0.301275550111244
CBWD5	0.488488467495326
CCDC110	-0.541356018245856
CCDC136	0.432022052205154
CCDC142	0.522109183076901
CCDC18	-0.435222816331576
CCDC34	-0.411215050215408
CCDC88A	-0.411446293094268
CCNA2	-0.426049411477204
CCNB1	-0.358405173720479
CCNK	-0.301470946541208
CCNL2	0.458271034015741
CCR1	0.232315087799115

CD81	0.270400171008454
CDC20	-0.317173728877022
CDC27	-0.397030225273344
CDC42BPG	0.334792152706721
CDC42EP1	-0.585223656732898
CDC42SE1	0.361819946739746
CDC73	-0.302684152015824
CDCA8	-0.365165979925011
CDK10	0.427753354967309
CDS2	0.400483799139984
CELSR1	0.36039849760049
CENPE	-0.686696757498597
CENPF	-0.512899942145811
CEP290	-0.408759409490187
CEP70	-0.445031435640694
CEP83	-0.348913903969911
CEP89	-0.438297894131131
CFL1	-0.53375453929266
CHML	-0.316087868375717
CIZ1	-0.395111743910098
CKAP2	-0.565421887439058
CKAP2L	-0.401191833655378
CKAP5	-0.426042465781282
CKLF	-0.419599284520201
CLIP1	-0.306056158792055
CLPX	-0.369870000029251
CLSPN	-0.317342674857771
CLSTN3	0.499882598998047
CNDP2	0.318009915176205
CNIH1	-0.259191972911471
CNN2	-0.341137314222195
CNOT1	-0.288088362478616
COL4A5	0.480472483083879
COPS2	-0.393755054300085
COX5A	-0.339295363743453
CPSF3	-0.287178854167598

CPVL	0.497225123557187
CREBBP	-0.322483555838808
CRLS1	0.374675743087039
CRTC2	-0.399187821309316
CRYL1	0.389069370052022
CSPP1	-0.321598635407746
CST6	0.473650228735522
CTNNAL1	-0.32407824138975
CUL2	-0.319321847713225
CUL5	-0.297453671379727
CYB5A	0.422439103631204
CYTH1	0.459743240713511
DAPK3	0.391552853160994
DCP1A	-0.289144857439998
DDOST	0.254552468434909
DDX46	-0.28636849492876
DEF8	0.349182750271111
DEK	-0.557289485625697
DEPDC1	-0.32855030166484
DFFB	0.544399203074556
DHRS7	0.324251207866305
DIAPH2	-0.385022008781051
DIAPH3	-0.451910787534047
DLAT	-0.276601004447642
DLGAP5	-0.480084433432068
DNAH2	0.418244451732089
DNAJC2	-0.427016019110963
DNAJC7	-0.327442843300729
DNMT1	-0.383839689152199
DTL	-0.28486161556883
DYNC1LI1	-0.30607373825598
EAPP	-0.341671751823468
ECT2	-0.431655422748784
EEF1A1	-0.650721524562345
EEF1D	-0.443391276504668
EFNA3	-0.431333806988828

EGR2	-0.505715010044182
EHF	0.592006368487107
EIF1	-0.343944827755148
EIF2A	-0.299320209284698
EIF3E	-0.326541832783698
EIF3J	-0.471042112911027
EIF3J-AS1	0.489960707567304
EIF4G2	-0.343520176014504
EIF4G3	-0.280172474260256
EIF5B	-0.419549782010073
ELMO3	0.404557048272354
ENAH	-0.251621032114413
ENDOV	0.44775779157414
ENSA	-0.398460963502978
EPB41L4A-AS1	0.412780592910666
ERC1	-0.244657124838244
ERCC6L	-0.351730911650621
ERCC6L2	-0.249351617449819
ERF	-0.571909888655751
ERMARD	0.433495605212496
EXTL3	0.30251157126476
EZH2	-0.296209362978077
FAM131A	0.428909683296446
FAM131C	-0.457865484682834
FAM198B	0.451796177758052
FAM210A	-0.402582334198828
FAM222B	-0.371427366569954
FAM46C	0.489892915078606
FARSB	-0.320250770433813
FBN3	0.574151846024106
FBXL6	0.649830426093248
FBXO5	-0.348497551499769
FER1L4	0.432005620788883
FGFRL1	-0.440159029962579
FGR	0.228661303241169
FKBP1A	-0.476181702832406

FKRP	0.478562531892004
FMNL3	-0.385913587681362
FN3K	0.428179243113226
FOSL1	-0.722157275314571
FOXM1	-0.448003037176379
FOXP4	-0.469863820836718
FXYD5	-0.397155823843482
GALK1	0.411873915969392
GAS2L3	-0.458734722370132
GATAD2B	-0.333897143607053
GCC2	-0.386722694506651
GDPD3	0.369479866139655
GJC1	-0.293279563339077
GLIS2	-0.387648640470041
GLUD1	-0.331168353914773
GLUD1P3	0.455660055971842
GNE	-0.391682339648885
GNG5	-0.352298528149856
GNS	0.27187694109709
GOLIM4	-0.4673373988081
GPATCH8	-0.300448123915629
GPR85	-0.637394714335659
GPR87	0.563499891144437
GPS2	-0.400107251644864
GPT2	0.404534956356711
GRIN2B	0.496900950527305
GSN	0.434415839369764
GTF3A	-0.312941895149043
GTPBP2	0.434055675774426
GUK1	0.380161084239288
HBB	-0.646568183513455
HCFC1R1	-0.60662562435138
HDAC2	-0.405308484812558
HELZ2	0.495702585624556
HIRA	0.378591498682525
HIST1H1E	-0.496395647086012

HIST1H2AJ	-0.411777747988852
HIST1H2BC	-0.497771461040435
HIST1H2BJ	-0.511731884964978
HIST1H2BK	-0.451997803936849
HIST1H2BO	-0.398315254282452
HIST1H3B	-0.416330567283081
HIST1H4E	-0.48049448279916
HIST2H2BE	-0.547077586779466
HIST2H2BF	-0.522018131963881
HJURP	-0.425443308349252
HMGA1	-0.407103257154261
HMGN5	-0.500686136662268
HMMR	-0.511815889099578
HNRNPC	-0.284041546860118
HNRNPUL1	-0.344925337971185
HOOK2	0.408047467469847
HOXA9	0.535323245718376
HOXB4	-0.442304183039474
HOXC9	-0.445296741813769
HSP90AA1	-0.570723073570974
HSPD1	-0.407281899363456
HUWE1	-0.318795450872057
IAH1	0.425453423055271
IBA57	0.527575800466288
IDE	-0.347262123260848
IER5	0.467577195735709
IGF2	-0.56940421007238
IL18	-0.557452881866994
IL4R	0.525733258689171
INCENP	-0.297859814203664
IPO7	-0.253720040366289
ITPKC	0.538911737915527
JADE1	-0.459769880738891
JAG1	0.38768730652108
KBTBD11	0.338007611972242
KHSRP	-0.48378643025521

KIAA0513	0.576426594430299
KIAA1430	-0.310103267566416
KIAA1524	-0.45401682959656
KIAA1551	-0.399362202819175
KIF11	-0.387028018247463
KIF14	-0.450594918705433
KIF15	-0.464405925697508
KIF18B	-0.438696515225161
KIF20B	-0.424983124415166
KIF23	-0.448781296400841
KIF4A	-0.426897277583648
KIF5B	-0.390571256695031
KIFAP3	-0.385949772491634
KLF2	-0.429108463791522
KLHL29	0.416839271466406
KTN1	-0.375673263266003
LAMB2	0.384296247428394
LENG1	-0.439113266723241
LEO1	-0.33138020964014
LHFP	0.340784096541479
LIN54	-0.332010791540861
LMTK3	-0.582904140472594
LNX1	0.471851216244092
LOC100129940	0.327211307731664
LOC100294362	0.450691594142501
LOC100506639	0.413789822244756
LOC729603	0.400758074599106
LRPPRC	-0.293235763571274
LRRC24	0.432528995836582
LRRC4	0.291747567600359
LYAR	-0.403112395578949
MAGED2	0.352019911329269
MAMDC2	0.455831191772148
MAP4	-0.399420869774726
MAP7D3	-0.497621336881016
MARCH2	0.497838052665334

MAT2A	0.390529418254092
MBNL1	-0.308266995311116
MESDC1	0.392016948217664
METAP2	-0.313385247031822
MGC57346	0.367420966769382
MIDN	-0.372680519636543
MIRLET7A1	-0.27078936243161
MIS18BP1	-0.510474884262151
MKI67	-0.746442145601877
MND1	-0.53967526500009
MNS1	-0.441025778488804
MOB1A	-0.290573385976552
MON1B	0.381303629141665
MPHOSPH10	-0.427394170448864
MRPL14	0.379058444111132
MUC16	-0.673019159421924
MYBL1	-0.522322903442785
MYEOV2	-0.382907501261218
MYL6	0.286007529219519
NAA15	-0.284925018219422
NAA20	0.455648951855004
NACA2	-0.361132339322295
NAF1	-0.313466931525455
NAP1L1	-0.397082976647118
NAPB	0.491901462577423
NAPRT1	0.521303863812991
NBPF1	0.443119644658794
NCAPG	-0.364335631429959
NCAPH	-0.450844402482461
NCL	-0.504947541292949
NCOA6	-0.29557118898387
NDUFA12	-0.434832377203934
NDUFAF5	0.473384906351344
NECAP2	0.44426338186956
NEK1	-0.302825819407015
NEK5	0.4317464786642

NEMF	-0.319627225122138
NFIC	-0.581779022478663
NIN	-0.252964071586836
NIPBL	-0.314451849994404
NKRF	-0.321106981477948
NME2	0.390088548573834
NOL7	-0.345746154901305
NOL8	-0.284820256383112
NOLC1	-0.33357927019218
NPHP3-ACAD11	0.314637490101937
NSFL1C	-0.317716054118973
NTRK2	0.29201505468139
NUDT22	0.417200682209591
NUDT3	-0.656434595390825
NUF2	-0.39104995045142
NUP153	-0.277153669114639
OAS2	0.419166644597332
OASL	0.441160681504732
OCLN	0.370911603374748
OGFOD3	0.451567967059753
OGFRL1	-0.410850499421936
OLA1	-0.378185181138914
ORMDL3	0.417455435156287
OTUD5	-0.527039447269328
PABPC1	-0.563918718676143
PABPC1L	0.46016553725137
PABPC1P2	-0.341124787142598
PARPBP	-0.430044322303456
PAWR	-0.340496971201139
PCDHB9	0.392291946713244
PCED1A	0.555340148763027
PCF11	-0.356911051650464
PCM1	-0.255177671753118
PDCD5	-0.392943142825545
PDF	0.488143032673022
PDS5A	-0.258121194618847

PELP1	-0.356414194003634
PFDN4	-0.483108211443989
PFN1	-0.732975544777461
PHC3	-0.235374158195469
PHIP	-0.370584474594911
PIAS1	-0.297842916222969
PIBF1	-0.560066458730872
PIK3AP1	0.38907298980863
PIP4K2A	-0.48935877472394
PITPNA-AS1	-0.416301261970958
PKD1	0.396495429682182
PKIB	0.513500448890105
PKP3	0.293603922358151
PLA2G15	0.480534548095628
PLA2G16	0.43161869739069
PLEKHG1	0.335841304753553
PLEKHO2	0.504639283523286
PLK1	-0.376212840556635
PMF1	-0.559022515131364
PMP22	-0.382078265195539
POLA1	-0.373718666668344
POMZP3	-0.519226784956395
PON2	0.354423930731305
PPAPDC1B	0.469889104069623
PPIC	0.505228076945847
PPIG	-0.450032605657474
PPP1R12A	-0.305095455959534
PPP1R15A	0.434062454588562
PPP1R3B	0.290004268553669
PPP3CA	-0.300817668338682
PRC1	-0.396551934460668
PRC1-AS1	0.278047642180813
PRKAA1	-0.301948282573971
PRKACA	-0.458527381873152
PRKCH	0.383698441094298
PRKDC	-0.404437159970673

PROM2	0.505848433056827
PRPF40A	-0.302788632222988
PRPF8	-0.291065582870919
PRR11	-0.535605269778802
PRRC2C	-0.436813336212401
PRSS3	0.411877124052163
PSIP1	-0.539573797400791
PSMD1	-0.305689022371644
PSMD6	-0.307570258458874
PTGES3	-0.289637495332961
PTMS	-0.508998247533719
PUS1	0.393847704112917
PVRL4	0.571708972373793
PVT1	-0.431221942486525
QSER1	-0.282886826979526
QSOX1	0.310126024680333
R3HDM1	-0.279323877806886
R3HDM2	-0.37371235555492
RAB25	-0.385431681090629
RABAC1	0.484489452055932
RAD18	-0.468644556358022
RAD21	-0.283132726469625
RAD50	-0.337546737061657
RAI14	-0.293887032466855
RALGAPA2	0.461699086147509
RASSF2	0.511943561044065
RASSF7	0.436533626415359
RAVER1	-0.373795536375669
RB1	-0.33191894146881
RBBP6	-0.320522027909834
RBM25	-0.410027929899303
RBM26	-0.341470105958788
RBMXL1	-0.400689942769738
RCAN2	0.5215014134892
RGS2	-0.402401574713202
RIF1	-0.296415159300606

RIPK4	0.45886138659255
RNA5-8S5	-0.267220145303479
RNF141	0.367217737565073
RNF219-AS1	0.274796146161212
RNF24	0.420538019959028
RNMTL1	0.382819581045015
RNY5	-0.293554306414785
RPAP3	-0.365656737041315
RPL26	-0.460939054845248
RPL29	-0.64603402093402
RPL30	-0.377245312521628
RPL32	-0.456476978578674
RPL35	-0.364622256077179
RPL37	-0.429370991291514
RPL39	-0.579448052707007
RPL4	-0.448128633360536
RPL41	-0.431012308667265
RPL6	-0.644036246263894
RPS15A	-0.4201925950298
RPS24	-0.347266496473355
RPS25	-0.476280389322799
RPS26	-0.422075705146259
RPS27A	0.39800803134978
RPS4X	-0.404086862716403
RPS9	0.360483860514492
RSAD2	0.795015998962265
RSF1	-0.468021317545739
RSL1D1	-0.387282801266688
RSL24D1	-0.307350079345054
RSRC2	-0.367045701452233
RTN3	-0.302152703025506
RTN4	-0.293339968801683
RUNX3	-0.430582415836125
S100A2	-0.505381938731971
S100A4	-0.481189544028263
S100A6	-0.607541893351658

SAR1A	0.260580393301399
SASS6	-0.46916963209558
SCAND1	0.460158021691315
SCARNA11	-0.386315371879617
SCARNA9	-0.50433703559419
SCARNA9L	-0.418316126392798
SCLT1	-0.41303422287227
SCYL3	0.450960804776446
SDAD1	-0.300230469604794
SDR42E1	0.386914440849196
SEC23B	0.339912927341773
SELO	0.405146355017674
SEMA3B	0.442394695154729
SEMA3D	0.413524321937729
SENP6	-0.281424258500433
SEPP1	0.464385815740201
SEPT10	-0.244057079044151
SEPT5	-0.354058350613448
SERBP1	-0.28181146230323
SETD1A	-0.409965331860565
SF3A1	-0.346722319924777
SF3A2	-0.463204643950997
SF3B1	-0.239035193044971
SF3B4	-0.401295664967023
SGOL2	-0.556530870274975
SH2D4A	-0.382544515333813
SH3BP5	0.311725353836101
SIK2	-0.308452179279213
SKA3	-0.537351857701794
SKIV2L2	-0.265051771531753
SLC15A4	0.375639953599511
SLC1A1	0.419502331612537
SLC25A23	-0.414169906561047
SLC25A29	0.467260805201676
SLC35B1	0.402969855091522
SLC35E2	0.445788415276851

SLC38A10	0.35761350339088
SLTM	-0.254976625765913
SMARCC1	-0.484704157738034
SMC2	-0.394577362436096
SMC3	-0.400444854535999
SMC4	-0.548738506861798
SMC6	-0.425727826866647
SMIM14	0.51460365728411
SMNDC1	-0.321270985623694
SMO	0.383872402899704
SNHG15	0.445500378188059
SNORA70	-0.423427755989491
SNORA71C	-0.459204417060777
SNORA76C	0.488258528062619
SNORA7B	-0.440517859682704
SNORA80B	-0.436153101284686
SNORA84	-0.397313547747333
SNORD100	-0.50574862617327
SNORD104	-0.535486471805568
SNORD105	-0.590405728891798
SNORD105B	-0.695913975125125
SNORD11	-0.629028286739052
SNORD119	-0.791837330127134
SNORD12	-0.567465358262408
SNORD12B	-0.602703506428129
SNORD1C	-0.623218094805933
SNORD21	-0.434968580475173
SNORD24	-0.658750634019142
SNORD27	-0.533049184351469
SNORD28	-0.428281917535574
SNORD30	-0.642566759785902
SNORD31	-0.666752889448958
SNORD36A	-0.675015288444612
SNORD36B	-0.44648655084991
SNORD36C	-0.515665400751476
SNORD37	-0.427706122782574

SNORD41	-0.695845520467241
SNORD42B	-0.494877346948506
SNORD44	-0.728873414007869
SNORD45A	-0.697429416697169
SNORD45C	-0.517184406532887
SNORD47	-0.649183231947748
SNORD54	-0.539060524467468
SNORD56	-0.706915303175169
SNORD57	-0.435420795556438
SNORD58A	-0.410388977859028
SNORD58C	-0.761530758019399
SNORD59B	-0.411879481657347
SNORD60	-0.449390875503887
SNORD61	-0.704952923685336
SNORD63	-0.472097186380742
SNORD65	-0.516440618729453
SNORD66	-0.519979106372791
SNORD69	-0.654698674656874
SNORD70	-0.563305673515902
SNORD76	-0.474288255212209
SNORD78	-0.50548577883421
SNORD80	-0.530986581951545
SNORD81	-0.496375819048478
SNORD87	-0.709828679240083
SNORD88A	-0.492294656247368
SNORD91A	-0.384166633530441
SNORD91B	-0.495089346599663
SNORD92	-0.482962340457219
SNRPA	-0.372125980456507
SNRPE	0.436736143633325
SNW1	-0.346473146601228
SORBS3	-0.514635309131399
SP2	-0.460959622402585
SP6	0.42393777272357
SPAG5-AS1	0.267466179002138
SPDL1	-0.412740702668143

SPINT2	0.338466677574938
SPPL2B	0.433342056553924
SPTB	0.493209243291898
SRPK1	-0.4060583349953
SRRM1	-0.378713307936089
SRSF5	0.427628095880655
SSRP1	-0.472993429005747
ST13	-0.444011442460024
ST14	0.379056174990382
ST3GAL3	-0.434096860274256
STAG1	-0.332624273296877
STAU1	-0.270101419802013
STIL	-0.370395428547316
STMN1	-0.392855469565996
STX16	0.440143045229362
STYXL1	-0.419620261240496
SUMF1	0.546168864290902
SUMO2	-0.542805604504468
SUPT6H	-0.280985155071849
SWAP70	-0.313095610350958
SYNCRIP	-0.259692206908298
SYNJ2	0.438811441365119
TACC2	-0.411122567043842
TAF3	-0.356195719474448
TAF4	-0.367277626737069
TAX1BP3	0.389873813688345
TBC1D16	0.448349861935676
TCF7L1	-0.511534041139219
TCP1	-0.327926237347428
TDRD3	-0.53530710130795
TFAP4	-0.430879307262994
TGFB2	-0.538784706124319
THBD	0.312490544632494
THBS1	-0.615154526476278
THBS3	0.535568398591169
THEMIS2	0.433496257671605

THOC7	-0.432481077182471
TIAL1	-0.320804882115106
TIMM9	-0.556724235297019
TLK1	-0.369522055484659
TM9SF1	0.343610908724865
TMED9	0.53669177103186
TMEM120B	0.475280958030546
TMEM123	-0.291568062211601
TMEM131	-0.342024873450406
TMEM139	0.427801378458936
TMEM179B	0.358936266207947
TMEM44	0.565440824572907
TMEM8A	0.484667520267198
TMEM98	0.513164485362764
TOB1	-0.419910108492419
TOP1	-0.405583365735689
TOP2A	-0.48255571794485
TP53I11	-0.429783810462506
TPM2	-0.411805744428881
TPM3	-0.430254062841265
TPR	-0.350999911478261
TPT1	-0.652600748702457
TPX2	-0.468372162992017
TRAPPC5	0.527656016104029
TSPAN10	-0.345491987970029
TSPAN13	0.48316551064215
TSPAN14	0.389140474270346
TSPAN31	0.417689945719787
TTC1	-0.337547582929519
TTK	-0.383584934112134
TUBA1B	-0.29852016764763
TUBB	-0.450008559909975
TUBB4A	-0.515318227908403
TXNL1	-0.433460941058712
U2SURP	-0.260632823614054
UACA	-0.453907666329128

UBAC2-AS1	0.441500644147032
UBALD1	-0.500843221595638
UBE2V2	-0.341731755019479
UBQLN1	-0.317826228059099
UBQLN2	-0.338677913133651
UBR3	-0.246350052036992
UCHL5	-0.302754020452681
UNC119B	0.603227592546756
USP10	-0.272507070037546
UTS2B	-0.509191099128417
VPS18	0.346845529585422
VRK1	-0.345762992492345
VTCN1	0.712181959041402
VTRNA1-2	0.31958086589279
VTRNA1-3	0.442771733692266
WAC	-0.255570876308219
WAPAL	-0.361487200840092
WBP11	-0.368007416783856
WBP2	-0.405529254259837
WDR4	0.627530792007874
WDR66	0.513849226045594
WNT10B	0.414485886979339
XPO1	-0.329530629984126
YBX1	-0.37720156630221
YWHAE	-0.428674256632632
YWHAEP1	-0.34378152542273
YWHAQ	-0.243524871916403
ZBED2	-100.725.121.096.312
ZBTB34	0.417315572024076
ZBTB4	-0.376738495567529
ZBTB7A	-0.522535384600072
ZC3H13	-0.46616663356783
ZC3H15	-0.317742314442393
ZCCHC10	-0.396868483869739
ZFP30	-0.457187750222499
ZIC2	0.404054277740159

ZMIZ2	-0.368348694211105
ZNF133	0.548925914059015
ZNF207	-0.258156286342458
ZNF263	0.340959484210055
ZNF276	0.440107052383951
ZNF385A	-0.612808400660952
ZNF385B	-0.437289577396025
ZNF423	0.449853472661985
ZNF432	0.386457689821558
ZNF579	-0.492568711927218
ZNF580	-0.47461841096824
ZNF589	0.466249841313642
ZNF608	0.370621731092185
ZNF624	-0.427394405209512
ZNF644	-0.401904895896494
ZNF696	0.426660632690652
ZNF704	0.474261061538553
ZNF707	0.508094221898776
ZNF776	0.437661444222065
ZNF783	0.453913830896684
ZNF800	-0.436172932690704
ZNF804A	0.430877525221433
ZNF808	0.385849241217813
ZNRF3	0.432017936894937
ZSCAN32	0.389408111920594

*Genes were listed with the exception of the 68 common genes reported in Figure 3c.
