# Supplementary material for: Overexpression of the cohesin-core subunit SMC1A contributes to colorectal cancer development
Source: J Exp Clin Cancer Res. 2019 Mar 1;38:108. doi: 10.1186/s13046-019-1116-0 (PMC6397456; doi:10.1186/s13046-019-1116-0)
Supplement: Supplementary file 13 — Figure S13. GO term enrichment analysis of biological processes that were significantly overrepresented when considering differentially expressed genes in HCT116 SMC1A wild-type cells. Figure S14. GO term enrichment analysis of biological process that were significantly overrepresented when considering differentially expressed genes in HCT116 SMC1A c.A2027G cells. Table S10. Dysregulated pathways identified in tumors induced by SMC1A wild-type with p < 0.01. Table S11. Dysregulated pathways identified in tumors induced by SMC1A c.A2027G with p < 0.01. (PDF 3535 kb) [file 13046_2019_1116_MOESM13_ESM.pdf]

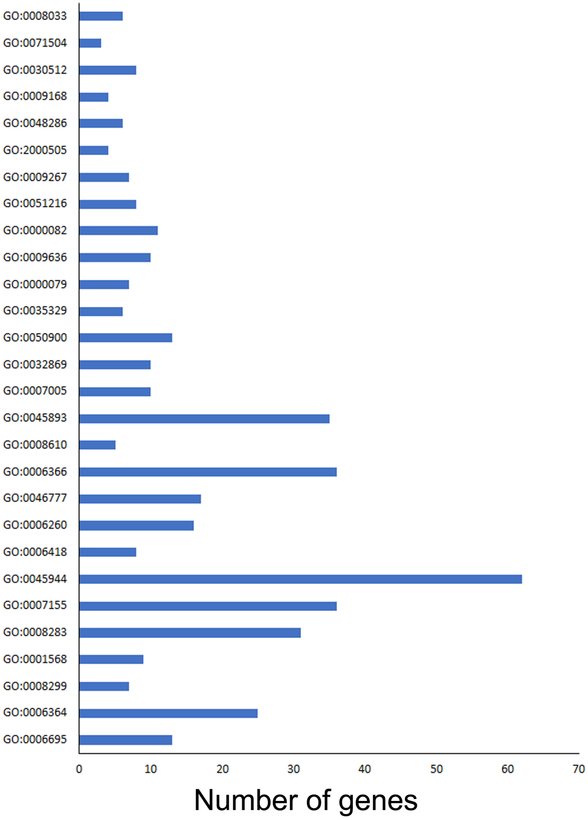

Supplementary Figure 13

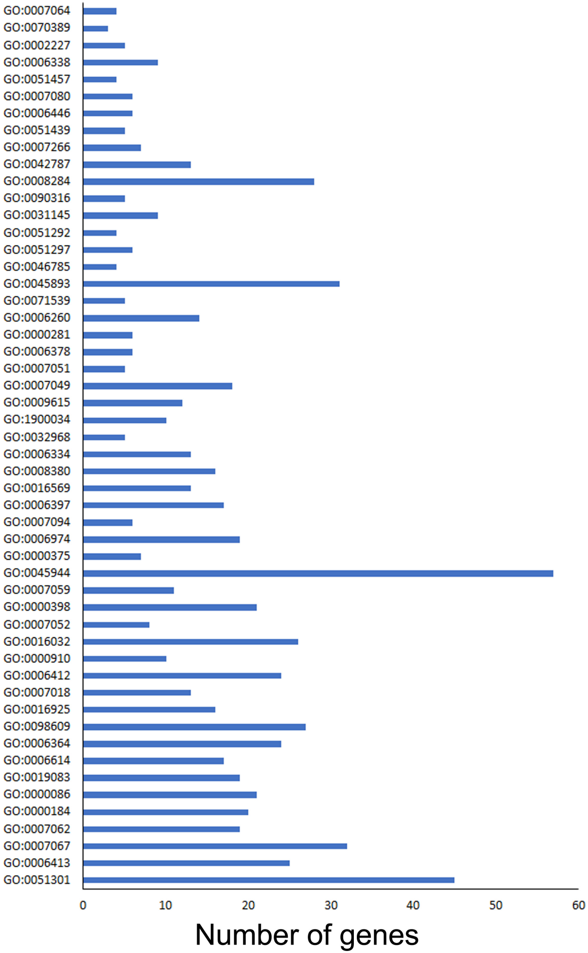

Supplementary Figure 14

Supplementary Table 10. Dysregulated pathways identified in tumors induced by *SMC1A* wild-type with p< 0.01.

**Pathway Description**	**P-value**
GO:0006695~cholesterol biosynthetic process	1,50E-08
GO:0006364~rRNA processing	5,00E-06
GO:0008299~isoprenoid biosynthetic process	9,00E-06
GO:0001568~blood vessel development	1,00E-04
GO:0008283~cell proliferation	1,60E-04
GO:0007155~cell adhesion	2,00E-04
GO:0045944~positive regulation of transcription from RNA polymerase II promoter	3,70E-04
GO:0006418~tRNA aminoacylation for protein translation	9,20E-04
GO:0006260~DNA replication	1,40E-03
GO:0046777~protein autophosphorylation	1,50E-03
GO:0006366~transcription from RNA polymerase II promoter	1,50E-03
GO:0008610~lipid biosynthetic process	1,80E-03
GO:0045893~positive regulation of transcription DNA-templated	2,90E-03
GO:0007005~mitochondrion organization	3,50E-03
GO:0032869~cellular response to insulin stimulus	3,50E-03
GO:0050900~leukocyte migration	3,50E-03
GO:0035329~hippo signaling	3,90E-03
GO:0000079~regulation of cyclin-dependent protein serine/threonine kinase activity	4,20E-03
GO:0009636~response to toxic substance	6,70E-03
GO:0000082~G1/S transition of mitotic cell cycle	7,50E-03
GO:0051216~cartilage development	8,80E-03
GO:0009267~cellular response to starvation	1,10E-02
GO:2000505~regulation of energy homeostasis	1,10E-02
GO:0048286~lung alveolus development	1,10E-02
GO:0009168~purine ribonucleoside monophosphate biosynthetic process	1,30E-02
GO:0030512~negative regulation of transforming growth factor beta receptor signaling pathway	1,40E-02
GO:0071504~cellular response to heparin	1,50E-02
GO:0008033~tRNA processing	1,50E-02

Supplementary Table 11. Dysregulated pathways identified in tumors induced by *SMC1A* c.A2027G with p< 0.01.

**Pathway Description**	**P-value**
GO:0051301~cell division	1,30E-13
GO:0006413~translational initiation	5,40E-11
GO:0007067~mitotic nuclear division	6,60E-10
GO:0007062~sister chromatid cohesion	1,50E-08
GO:0000184~nuclear-transcribed mRNA catabolic process nonsense-mediated decay	2,70E-08
GO:0000086~G2/M transition of mitotic cell cycle	5,50E-08
GO:0019083~viral transcription	5,60E-08
GO:0006614~SRP-dependent cotranslational protein targeting to membrane	1,40E-07
GO:0006364~rRNA processing	1,60E-06
GO:0098609~cell-cell adhesion	3,00E-06
GO:0016925~protein sumoylation	1,30E-05
GO:0007018~microtubule-based movement	2,20E-05
GO:0006412~translation	2,60E-05
GO:0000910~cytokinesis	3,40E-05
GO:0016032~viral process	4,80E-05
GO:0007052~mitotic spindle organization	6,00E-05
GO:0000398~mRNA splicing via spliceosome	9,80E-05
GO:0007059~chromosome segregation	1,10E-04
GO:0045944~positive regulation of transcription from RNA polymerase II promoter	1,70E-04
GO:0000375~RNA splicing via transesterification reactions	1,70E-04
GO:0006974~cellular response to DNA damage stimulus	3,50E-04
GO:0007094~mitotic spindle assembly checkpoint	5,00E-04
GO:0006397~mRNA processing	5,20E-04
GO:0016569~covalent chromatin modification	5,60E-04
GO:0008380~RNA splicing	6,80E-04
GO:0006334~nucleosome assembly	8,80E-04
GO:0032968~positive regulation of transcription elongation from RNA polymerase II promoter	1,10E-03
GO:1900034~regulation of cellular response to heat	1,10E-03
GO:0009615~response to virus	1,50E-03
GO:0007049~cell cycle	1,50E-03
GO:0007051~spindle organization	1,90E-03
GO:0006378~mRNA polyadenylation	2,50E-03
GO:0000281~mitotic cytokinesis	2,90E-03
GO:0006260~DNA replication	3,00E-03
GO:0071539~protein localization to centrosome	3,00E-03
GO:0045893~positive regulation of transcription DNA-templated	4,10E-03
GO:0046785~microtubule polymerization	4,20E-03
GO:0051297~centrosome organization	4,80E-03

GO:0051292~nuclear pore complex assembly	5,30E-03
GO:0031145~anaphase-promoting complex-dependent catabolic process	6,00E-03
GO:0090316~positive regulation of intracellular protein transport	6,40E-03
GO:0008284~positive regulation of cell proliferation	6,70E-03
GO:0042787~protein ubiquitination involved in ubiquitin-dependent protein catabolic process	7,10E-03
GO:0007266~Rho protein signal transduction	7,40E-03
GO:0051439~regulation of ubiquitin-protein ligase activity involved in mitotic cell cycle	7,50E-03
GO:0006446~regulation of translational initiation	7,70E-03
GO:0007080~mitotic metaphase plate congression	8,60E-03
GO:0051457~maintenance of protein location in nucleus	9,20E-03
GO:0006338~chromatin remodeling	9,90E-03
GO:0002227~innate immune response in mucosa	1,00E-02
GO:0070389~chaperone cofactor-dependent protein refolding	1,10E-02
GO:0007064~mitotic sister chromatid cohesion	1,10E-02
